# Melatonin-incorporated brain extracellular matrix hydrogel enhances NSCs mitochondrial metabolism to promote neuroregeneration via the AMPK-PGC-1α-NRF1/TFAM axis after spinal cord injury

**DOI:** 10.1016/j.bioactmat.2026.04.006

**Published:** 2026-04-09

**Authors:** Rushuo Wei, Quanjing Mei, Tiangang Zhou, Xiaoqian Zhang, Weiqiang Liu, Mingdong Yu, Bingwu Wang, Hui-Qi Xie, Ruzhan Yao

**Affiliations:** a Department of Spinal Surgery, Weifang People's Hospital, Shandong Second Medical University, Weifang, Shandong, 261000, China; b Digital Spine and Minimally Invasive Research Institute, Shandong Second Medical University, Weifang, Shandong, 261000, China; c Shandong Provincial Key Medical and Health Laboratory of orthopedic Rare Diseases Prevention and Digital Technique Medicine-engineering Transformation, Weifang People's Hospital, Shandong Second Medical University, Weifang, Shandong, 261000, China; d Department of Orthopedic Surgery and Orthopedic Research Institute, Stem Cell and Tissue Engineering Research Center, State Key Laboratory of Biotherapy, West China Hospital, Sichuan University, Chengdu, 610041, Sichuan, China

**Keywords:** Spinal cord injury, Brain extracellular matrix hydrogel, Neural stem cells, Melatonin, AMPK signaling

## Abstract

Spinal cord injury (SCI) results in severe and debilitating neurological damage. Mitochondria play a crucial role in the differentiation of neural stem cells (NSCs) and neural regeneration. However, mitochondrial dysfunction occurs following SCI, manifesting as dysregulation of oxidative stress and ATP depletion, which impede neural regeneration. In this study, we developed a multi-functional, injectable hydrogel platform by integrating a brain-derived extracellular matrix (BEM), the neuroprotective agent melatonin (MT), and NSCs. We hypothesized that this NSCs@MT/BEM system would create a synergistic niche in which BEM provided tissue-specific signals, and melatonin metabolically reprogramed NSCs to enhance the regenerative potential. In vitro, melatonin directed NSCs differentiation towards a neuronal fate by enhancing mitochondrial function through AMPK signaling. When transplanted into a rat model of thoracic spinal cord contusion, the NSCs@MT/BEM hydrogel elicited robust functional recovery, evidenced by significantly improved Basso-Beattie-Bresnahan (BBB) scores, restored gait patterns, and enhanced electrophysiological conduction. This functional repair was supported by profound structural changes: enhanced survival of transplanted NSCs, preserved host neurons, attenuated glial scarring, and robust serotonergic axon regeneration across the lesion. Our findings demonstrated that a strategy combining a biomimetic scaffold with targeted metabolic modulation created a pro-regenerative microenvironment, significantly enhancing the therapeutic efficacy of NSCs transplantation for SCI repair.

## Highlights

• Brain extracellular matrix hydrogel co-delivers melatonin and neural stem cells.
• Melatonin activates AMPK to boost NSCs mitochondrial function and neuronal fate.
• NSCs@MT/BEM improves BBB scores, gait metrics, and MEPs after thoracic SCI.
• Therapy enhances graft survival, preserves neurons, and reduces glial scarring.
• Promotes serotonergic axon regeneration across the lesion for functional recovery.

## Graphical abstract

**Figure:**
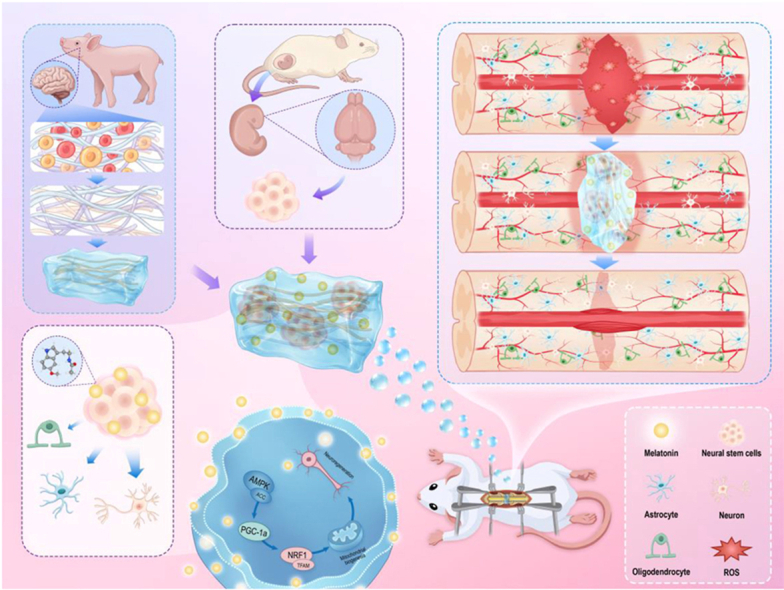

## 1 Introduction

Spinal cord injury (SCI) is a devastating neurological condition characterized by the partial or complete loss of motor, sensory, and autonomic functions below the injury site. While neural stem cells (NSCs) based therapy has emerged as a promising frontier for spinal repair-owing to its potential for neuronal differentiation and neural circuit reconstruction-its therapeutic efficacy is fundamentally constrained by the hostile oxidative microenvironment. Pathologically, SCI is initiated by irreversible primary damage from mechanical forces, followed by the triggering of secondary injury through a cascade of reactions, including local hemorrhage, ischemia-hypoxia, ionic imbalance, and free radical stress [1,2]. Particularly, oxidative stress resulting from excessive reactive oxygen species production acts as a key driver for the deterioration of the pathological microenvironment, thereby profoundly impeding the survival and regenerative potential of NSCs [3,4].

Mitochondria, the energy hub of the cell, play a critical role in neural regeneration by regulating the fate of NSCs and provide energy as well as biosynthetic materials for axonal regeneration [5]. Mitochondria are particularly vulnerable to this oxidative damage, and their subsequent dysfunction is a critical determinant of neuronal survival and repair following SCI [6]. These compromised mitochondria fail to produce sufficient ATP but become a significant source of ROS instead, creating a vicious cycle that perpetuates cellular injury. Impaired mitochondrial function not only compromises the survival and proliferation of NSCs but also suppresses neurogenesis, leading to excessive astrocyte production and thereby severely hindering neural regeneration. Hence, the design of advanced delivery systems capable of actively modulating the post-injury microenvironment-particularly by attenuating oxidative stress and restoring mitochondrial function-represents a highly promising therapeutic avenue. Such systems are essential for sustaining NSCs proliferation and steering lineage-specific differentiation into neurons, which collectively contribute to functional recovery after SCI.

Melatonin (N-acetyl-5-methoxytryptamine), a hormone primarily secreted by the pineal gland, has been extensively implicated in the regulation of cellular processes including differentiation, apoptosis, and inflammatory signaling pathways [7,8]. Importantly, melatonin and its metabolites can reduce neuronal death through antioxidant responses. Accumulating evidence further demonstrates that melatonin promotes neurogenesis of NSCs by upregulating the expression of mature neuronal markers while inhibiting the expression of the astrocytic marker glial fibrillary acidic protein (GFAP) [9,10]. Notably, mitochondria constitute the primary intracellular target of melatonin. By maintaining the efficiency of oxidative phosphorylation and ATP synthesis and enhancing the activity of respiratory chain complexes, melatonin is considered a potential regulator of mitochondrial function [11]. Furthermore, its regulatory effects can prevent damage to mitochondrial respiratory function [12,13]. Despite these advances, the role of melatonin at the mitochondrial level during the differentiation of neural stem cells in spinal cord injury has not yet been fully elucidated. Moreover, the therapeutic efficacy of both melatonin and NSCs transplantation is substantially limited by the short biological half-life of melatonin, as well as the poor engraftment, survival, and functional integration of transplanted NSCs within the ischemic, inflammatory, and oxidative SCI microenvironment.

In this context, hydrogel-based biomaterials have garnered increasing attention for spinal cord injury regeneration owing to their tissue-mimetic properties and versatile drug-loading capabilities. By recapitulating key biochemical and biomechanical features of native spinal cord extracellular matrix, hydrogels provide a supportive three-dimensional microenvironment that enhances cell retention and survival, while enabling the sustained and localized delivery of therapeutic agents. Despite these advantages, traditional implantable hydrogels often lack the structural resilience to withstand physiological mechanical stresses, rendering them susceptible to fragmentation and collapse [[14], [15], [16]]. Such structural failure not only triggers the non-specific infiltration of inflammatory cells-exacerbating local inflammation-but also poses a formidable challenge to achieving stable tissue-biomaterial integration.

Compared to conventional synthetic or single-component natural hydrogels (e.g., alginate, gelatin, PEG-based systems), decellularized extracellular matrix (ECM) are particularly advantageous, as they offer tissue-specific biochemical cues and biophysical characteristics commensurate with the host for cell 'reading and remodeling' [17]. Among extracellular matrices derived from different tissue sources, brain-derived extracellular matrix (BEM) was selected for this study due to its CNS-specific basement membrane components (such as high contents of HA and GAGs), and retention of key bioactive molecules, including brain-derived neurotrophic factor (BDNF) and nerve growth factor (NGF) [18]. Distinguished from other biomaterials, it more accurately replicates the native microenvironment that supports spinal cord repair. This capability fosters robust integration and stable fusion with the host spinal cord, which in turn enhances the process of neural regeneration. Furthermore, the brain offers practical advantages over the spinal cord due to its larger tissue volume and greater structural uniformity, enabling reproducible extraction of sufficient ECM for hydrogel preparation.

Building on these considerations, we developed a MT/BEM hydrogel capable of closely mimicking the spinal cord microenvironment for SCI repair. This hydrogel system exhibits multiple functionalities, including excellent injectability, rapid in situ gelation, sustained drug release and high bioactivity. Notably, the NSCs@MT/BEM hydrogel presents distinct advantages over traditional hydrogels: it not only possesses biological and mechanical properties akin to native spinal tissue—enabling efficient and stable integration—but also modulates the injurious microenvironment by reducing oxidative stress, enhancing mitochondrial function, and subsequently fostering NSCs neurogenesis. We therefore hypothesized that combining melatonin with a BEM hydrogel would synergistically modulate the post-SCI microenvironment by suppressing oxidative stress, enhancing mitochondrial function, and promoting neuronal differentiation of NSCs, thereby improving functional recovery. Our work aims to establish a basis for a clinically translatable, biomaterial-based melatonin delivery platform for SCI repair.

## 2 Materials and methods

### 2.1 NSCs culture

NSCs were isolated from embryonic day 16-18 Sprague-Dawley (SD) rat fetuses. Pregnant dams were euthanized by cervical dislocation, and the uteri were aseptically removed. Following disinfection in 75% ethanol, the embryos were dissected in D-Hank's solution. The brains were harvested, and the meningeal tissues were carefully removed. The cerebral cortex and hippocampus were then isolated on ice, minced, and dissociated into a single-cell suspension by passing the tissue through a 70 μm cell strainer. After centrifugation, the cell pellet was resuspended in serum-free NSCs culture medium, consisting of DMEM/F12 (GIBCO, C11330500BT, USA) supplemented with 2% B27 (GIBCO, 12587010, USA), 1% penicillin-streptomycin (HYCLONE, SV30010, USA), 20 ng/mL basic fibroblast growth factor (bFGF; MCE, HY-P7004, China), and 20 ng/mL epidermal growth factor (EGF; MCE, HY-P7109, China). Cells were seeded into 75 cm^2^ culture flasks at a density of 5-10 × 10^5^ cells/mL.

The culture medium was replaced every 2-3 days, and neurospheres typically formed within 6-7 days. Passaging was conducted when spheres reached a sufficient size, characterized by a dark center and a bright periphery. For the first passage (P1), spheres were mechanically dissociated; subsequent passages used enzymatic digestion with Accutase (GIBCO, A1110501, USA). Briefly, the neurosphere suspension was centrifuged at 600 rpm for 1 min. The cell pellet was then washed with PBS, centrifuged again, and the supernatant was discarded. Finally, the cells were incubated in Accutase at 37 °C for 8 min, gently pipetted to form a single-cell suspension, and reseeded into new 75 cm^2^ culture flasks in fresh medium. Representative immunofluorescence images demonstrating the identification of cultured NSCs using Nestin staining (Fig. S1).

NSCs were transduced with an EGFP-P2A-Puro lentiviral vector (MOI = 40) and subsequently selected with puromycin. The GFP-positive rate was ∼80% as assessed by fluorescence microscopy. GFP labeling was used to enable in vivo tracking of transplanted NSCs in subsequent animal experiments.

### 2.2 Cell viability assay

To assess the effects of melatonin on neural stem cells (NSCs), cell viability was evaluated using a Calcein-AM/PI Live/Dead Cell Double Staining Kit (CA1630, Solarbio, Beijing, China). Following exposure to different concentrations of melatonin, the NSCs were stained according to the manufacturer's protocol. The cells were then imaged using a fluorescence microscope (IX73, Olympus, Tokyo, Japan) to visualize live (green) and dead (red) cells. Cell morphology was also observed during imaging to evaluate the general biocompatibility of the treatment.

### 2.3 Reactive oxygen species (ROS) detection

NSCs were seeded in 6-well plates at a density of 2 × 10^5^ cells/well. The cells were then induced to differentiate by culturing them in DMEM/F12 medium containing 10% fetal bovine serum and varying concentrations of melatonin. Following the treatment period, intracellular reactive oxygen species (ROS) levels were measured using a DCFH-DA fluorescent probe (10 μM; S0033, Beyotime, China). The cells were incubated with the probe solution (1 mL/well) for 20 min at 37 °C in the dark, then washed three times with PBS. Fluorescence images were subsequently captured using an Olympus microscope (IX73, Olympus, Tokyo, Japan), and the mean fluorescence intensity was quantified with ImageJ software (v1.53, National Institutes of Health, USA).

### 2.4 Mitochondrial function assessment

For AMPK inhibition experiments, BAY-3827 (HY-112083, MedChemExpress, USA), a selective AMPK inhibitor, was used at a final concentration of 2 μM for 24 h. The mitochondrial membrane potential was measured using the JC-1 Mitochondrial Membrane Potential Assay Kit (C2003S, Beyotime Biotechnology, China). For the assay, cells were incubated with the JC-1 dye and then visualized with an Olympus fluorescence microscope (IX73, Olympus, Tokyo, Japan). Per the assay's principle, a decrease in mitochondrial membrane potential is indicated by green fluorescence, while normal membrane potential is represented by red fluorescence. To quantify this effect, the fluorescence intensity ratio was calculated in ImageJ (Version 1.53, National Institutes of Health, USA) to assess mitochondrial function.

In addition, ATP levels were measured using the ATP Assay Kit (S0026, Beyotime Biotechnology, China). Cells or tissue samples were lysed, and ATP concentrations were measured using a luminometer. The chemiluminescence produced by the reaction between firefly luciferase and luciferin was used to quantify ATP levels. Samples were prepared under ice conditions to maintain ATP stability, and the reaction was completed within 30 min for accurate readings. The results were expressed as relative ATP levels (fold-change vs Control) to facilitate cross-group comparisons and statistical analysis.

### 2.5 Reverse transcription and quantitative real-time PCR (RT-qPCR)

Total RNA was extracted from cells using the Eastep Super Total RNA Extraction Kit (LS1040, Shanghai Promega, China) according to the manufacturer's protocol. First-strand cDNA was then synthesized from the extracted RNA using the PrimeScript Fast RT Reagent Kit with gDNA Eraser (RR092A, Takara, Japan). Quantitative PCR was subsequently performed using TB Green Premix Ex *Taq*II Fast (CN830A, Takara, Japan) on a QuantStudio 5 real-time PCR system (Applied Biosystems, USA). The thermal cycling protocol was as follows: an initial denaturation at 95 °C for 30 s, followed by 40 cycles of denaturation at 95 °C for 5 s and annealing/extension at 60 °C for 10 s. After amplification, a melt curve analysis (65-95 °C) was conducted to confirm product specificity. Gene expression levels were normalized to the reference gene GAPDH, and relative expression was calculated using the 2^^^−ΔΔCt method. The sequences of all primers used in this study are listed in Table S1.

### 2.6 Western blot analysis

Protein lysates were prepared from cells or tissues using RIPA buffer (P0013B, Beyotime, China) supplemented with a protease and phosphatase inhibitor cocktail (P1051, Beyotime, China). The total protein concentration of each lysate was determined using a BCA assay kit (Beyotime, China). SDS-PAGE separated equal amounts of protein from each sample and subsequently transferred them onto polyvinylidene fluoride (PVDF) membranes (Millipore, USA). The membranes were blocked for 1 h at room temperature in TBST containing 5% non-fat milk (or 5% BSA for detecting phosphorylated proteins). Following blocking, membranes were incubated overnight at 4 °C with the following primary antibodies: GFAP (1:1000; #80788, CST, USA), TUJ1 (1:1000; #YA586, MCE, China), GAPDH (1:1000; #2118, CST, USA), phospho-ACC (Ser79) (1:1000; #11818, CST, USA), Acetyl-CoA Carboxylase (C83B10) (1:1000; #3676, CST, USA), Total OXPHOS Rodent WB Antibody Cocktail (1:1000; #ab110413, Abcam, UK), phospho-AMPKα (Thr172) (1:1000; #2535, CST, USA), and total AMPKα (1:1000; #2532, CST, USA). After being washed with TBST, the membranes were incubated with HRP-conjugated secondary antibodies (A0210 and A0218, Beyotime, China) for 1 h at room temperature. Protein bands were visualized with an ECL reagent (P0018, Beyotime, China) and imaged on a Tanon 5200 chemiluminescence system (Tanon, China).

### 2.7 RNA sequencing and bioinformatics analysis

Total RNA was extracted from NSCs samples using TRIzol reagent (15596026, Invitrogen, USA) according to the manufacturer's protocol. RNA sequencing libraries were constructed with the NEBNext Ultra Directional RNA Library Prep Kit for Illumina (New England Biolabs, USA). The libraries were then sequenced on an Illumina platform by Novogene (Beijing, China) to generate paired-end reads. For the bioinformatics analysis, raw reads were first subjected to quality control and adapter trimming. The processed reads were then aligned to the rat reference genome mRatBN7.2 (Ensembl release 110) using HISAT2 (v2.0.5). Gene-level read counts were subsequently generated using featureCounts (v1.5.0-p3). Differentially expressed genes were identified, with statistical significance corrected for multiple testing using the Benjamini-Hochberg (BH) method. Finally, to explore the biological roles of these genes, functional enrichment analyses were performed using the Gene Ontology (GO) and Kyoto Encyclopedia of Genes and Genomes (KEGG) databases.

### 2.8 Immunofluorescence staining

For immunofluorescence staining, cells were fixed in 4% paraformaldehyde (PFA) for 10 min, then washed three times with PBS. Permeabilization and blocking were performed concurrently by incubating the cells for 1 h at room temperature in PBS containing 0.3% Triton X-100 and 5% normal goat serum. Subsequently, the cells were incubated overnight at 4 °C with the following primary antibodies: mouse anti-Nestin (1:500, #89529, CST, USA), rabbit anti-GFAP (1:500, #3670, CST, USA), rabbit anti-Olig2 (1:500, #65915, CST, USA), and rabbit anti-TUJ1/β3-Tubulin (1:500, #5568, CST, USA). The following day, cells were washed three times with PBS before incubation with the appropriate fluorescently conjugated secondary antibodies for 2 h at room temperature. The secondary antibodies used included Alexa Fluor 488-conjugated goat anti-mouse IgG (1:200; A0428, Beyotime, China), Cy3-conjugated goat anti-rabbit IgG (1:200, A0516, Beyotime, China), Alexa Fluor 488-conjugated goat anti-rabbit IgG (1:200, A0423, Beyotime, China), and Cy3-conjugated goat anti-mouse IgG (1:200, A0507, Beyotime, China). Finally, after three additional PBS washes, cell nuclei were counterstained with DAPI. Fluorescence images were acquired using either a Leica SP8 confocal laser scanning microscope (Leica Microsystems, Germany) or a Celldiscoverer 7 automated imaging system (ZEISS, Germany).

### 2.9 Preparation of BEM

Fresh porcine brains (from 6-month-old pigs, processed <6 h post-mortem) were obtained from a local abattoir. After removing the pia mater, the brain tissue was cut into approximately 1 cm^3^ cubes and soaked in ultrapure water at 4 °C for 12 h. The tissue cubes were then sequentially decellularized at 4 °C under gentle agitation. This process involved serial incubations in 3% Triton X-100 (Biosharp, BS084, China; 90 min), 0.001% DNase I (Sigma-Aldrich, USA; 30 min), 1 M sucrose (Beyotime, ST1670, China; 30 min), 3% sodium dodecyl sulfate (SDS; Beyotime, ST625, China; 60 min), and 1% penicillin-streptomycin (Hyclone, SV30010, USA; 60 min). The entire decellularization process was conducted under sterile conditions at 4 °C to minimize endotoxin contamination. Following each treatment step, the tissues were rinsed three times with ultrapure water. To ensure efficient removal of residual detergents, the decellularized brain matrices were further washed in large volumes of deionized water for 48-72 h with frequent solution exchange (every 6-8 h). The resulting decellularized samples were snap-frozen at −80 °C, lyophilized for 24-48 h to obtain BEM.

After lyophilization, the BEM was cut into small pieces and ground using a cryogenic mixed grinder (MM400; Retsch, Germany). The resulting BEM powder was then digested with 0.1% (w/v) pepsin (Sigma, USA) in 0.01 M HCl (pH 2.0) for 48 h at 25 °C, followed by a second lyophilization. The lyophilized BEM was further sterilized using ethylene oxide gas sterilization. To prepare BEM hydrogels at concentrations of 1%, 2%, and 3% (w/v), 10, 20, and 30 mg of the sterilized BEM powder were respectively dissolved in DMEM/F12 medium (Gibco, 11320-033, USA), and the solution was subsequently neutralized with 0.1 M NaOH to adjust the pH to 7.0.

### 2.10 Hydrogel characterization

The microstructure of the MT/BEM hydrogel was examined by scanning electron microscopy (SEM; GeminiSEM 300, Zeiss, Germany). For analysis, samples were freeze-dried, sputter-coated with gold, and imaged at 20 kV and 200 × magnification. The hydrogel's viscoelastic properties were characterized using a rotational rheometer (MCR302, Anton Paar, Austria) equipped with a 25 mm parallel plate geometry at 37 °C. Oscillatory measurements were used to determine the storage modulus (G′) and loss modulus (G″), which represent the material's elastic and viscous components, respectively. Frequency sweep tests were performed over the range from 0.1 to 10 Hz at a constant strain of 1%, which lies within the linear viscoelastic region. All rheological measurements were performed with three independent samples (n = 3) under identical experimental conditions. The chemical structure of the hydrogels was characterized using a Fourier transform infrared (FTIR) spectrometer (Thermo, 6700, US). Spectra were recorded over a wavenumber range of 4000 cm^−1^ to 500 cm^−1^ with a resolution of 4 cm^−1^. For histological analysis, hydrogel sections were stained with a Modified Masson's Trichrome Stain Kit (G1346, Solarbio, China) to assess collagen distribution and extracellular matrix organization.

### 2.11 SCI model and experimental groups

A total of 55 female Sprague-Dawley rats (8-10 weeks old; 220-250 g) were obtained from Shandong Pengyue Laboratory Animal Technology and housed under specific-pathogen-free (SPF) conditions with a 12-h light/dark cycle. Animals were randomly allocated to five groups (n = 11 per group): Sham, SCI, BEM, NSCs@BEM, and NSCs@MT/BEM. This stepwise in vivo design enabled evaluation of incremental therapeutic gains from BEM (BEM vs SCI), from NSC loading within BEM (NSCs@BEM vs BEM), and from melatonin incorporation beyond the BEM–NSCs construct (NSCs@MT/BEM vs NSCs@BEM). All rats were administered ceftazidime (10 mg/kg, intraperitoneally; HY-B0593, MCE, USA) once daily, starting one day before surgery and continuing for 7 days postoperatively.

Surgical procedures were performed under isoflurane anesthesia, with body temperature maintained with a heating pad. A laminectomy was performed at the T9-T10 vertebral level to expose the spinal cord. In all groups except the Sham group, a moderate contusive injury was induced using a spinal cord impactor (RWD, China) with a 2-mm-diameter tip (impact parameters: 2 m/s velocity, 0.8 mm displacement, 0.5 s dwell time). Immediately after injury, animals in the treatment groups received a 5 μL injection of pre-chilled (4 °C) hydrogel solution into the lesion epicenter. The compositions were as follows: BEM hydrogel alone (BEM group); BEM hydrogel containing NSCs (5 × 10^4^ cells/μL) (NSCs@BEM group); or BEM hydrogel containing both NSCs (5 × 10^4^ cells/μL) and melatonin (50 μM) (NSCs@MT/BEM group) to evaluation of each treatment's incremental contribution. GFP-labeled NSCs were used in both cell-treated groups (NSCs@BEM and NSCs@MT/BEM) and were encapsulated in the BEM hydrogel (with or without melatonin) prior to intralesional injection. After allowing at least 3 min for in situ gelation, ensuring the hydrogel had solidified, the wounds were then surgically closed. All animals recovered uneventfully, and no perioperative mortality or animal exclusions occurred. Postoperatively, manual bladder expression was performed twice daily for all injured animals until spontaneous voiding function returned.

### 2.12 Behavioral assessment

The period of 4 to 8 weeks after spinal cord injury is considered the subacute phase, during which inflammatory responses gradually subside and damaged nerves begin to repair. Since this study primarily focuses on observing neural regeneration, the 8-week time point was selected [19]. First, the Basso-Beattie-Bresnahan (BBB) open-field test was performed. A detailed gait analysis was conducted using a footprint test. Animals were pre-trained on a small animal treadmill before the final gait analysis, which was conducted in a narrow acrylic corridor lined with white paper. Immediately prior to each test, the rats' forepaws were inked black and their hindpaws red. The captured footprints were then scanned and quantified using ImageJ (v1.53) for the following parameters: (i) Hindlimb Stride Length, the distance between successive prints of the same hindpaw; (ii) Hindlimb Base of Support, the perpendicular distance between left and right hindpaw prints; and (iii) Fore-hind Coordination, the distance between a forepaw print and the subsequent placement of the ipsilateral hindpaw. For each time point, a minimum of three consecutive steps per animal were measured and averaged. To minimize potential bias, all behavioral assessments, including BBB scoring and footprint-based gait analysis, were performed by assessors who were blinded to group allocation. This ensured that the evaluation of the animals’ performance was not influenced by group assignment.

### 2.13 Electrophysiological evaluation

To assess the electrical signal conduction, motor-evoked potentials (MEPs) were recorded. During the procedure, animals were kept under isoflurane anesthesia and their body temperature was maintained on a heating pad. The MEP measurements were performed by an investigator who was blinded to the group allocation to minimize potential bias. Using a stereotaxic frame, small burr holes were drilled in the skull over the motor cortex. Insulated bipolar stimulating electrodes were then stereotactically lowered to the motor cortex and secured to the skull with dental cement. The sciatic nerve was surgically exposed at the mid-thigh, and a bipolar hook electrode was placed around the nerve. A reference electrode was positioned in the adjacent subcutaneous tissue, and a ground electrode was placed subcutaneously over the back.

Cortical stimulation was delivered as a series of 2 mA pulses at a frequency of 10 Hz. The resulting MEP waveforms were continuously acquired from the sciatic nerve. For data analysis, five non-overlapping MEP wave groups were randomly selected from each animal's recording. The peak-to-peak amplitude (calculated as the maximum positive peak minus the minimum negative peak) was determined for each wave group. The final reported value for each animal was the mean of these five amplitude measurements. Representative waveforms were selected for figures.

### 2.14 Histology and immunofluorescence staining (spinal cord tissue)

For endpoint histological analysis, rats were deeply anesthetized with isoflurane and transcardially perfused, first with ice-cold PBS until the liver visibly blanched, followed by ice-cold 4% PFA. The spinal cords were carefully harvested by laminectomy, post-fixed in 4% PFA for 24 h, and then cryoprotected by immersion in a sucrose gradient. Subsequently, the tissue segments were embedded in OCT compound, and 8 μm-thick cryosections were prepared using a cryostat (CT520, Dakewe, China).

Basic tissue morphology was assessed with a standard Hematoxylin-Eosin (H&E) staining kit (G1120, Solarbio, China). For immunofluorescence, frozen sections were brought to room temperature for 15 min, washed with PBS to remove residual OCT, and post-fixed in 4% PFA for 15 min. A hydrophobic barrier was drawn around the tissue. Sections were then permeabilized with 0.3% Triton X-100 in PBS and blocked with 5% normal goat serum for 1 h at room temperature. The sections were subsequently incubated overnight at 4 °C with the following primary antibodies: mouse anti-NeuN (1:500; ab104224, Abcam, UK), rabbit anti-NF200 (1:500; ab8135, Abcam, UK), rabbit anti-GFAP (1:500; #3670, CST, USA), rabbit anti-Olig2 (1:500; #65915, CST, USA), rabbit anti-5-HT3A receptor (1:500; ab271031, Abcam, UK), and rabbit anti-GFP (1:500; #2555, CST, USA), which was used exclusively for the NSCs@BEM and NSCs@MT/BEM groups. After washing, sections were incubated for 1-2 h in the dark at room temperature with the appropriate secondary antibodies (Alexa Fluor 488-conjugated goat anti-mouse, A0428, Beyotime; or Cy3-conjugated goat anti-rabbit, A0516, Beyotime). Following final washes, the sections were mounted with an antifade medium containing DAPI (F6057, Abcam, UK) to counterstain cell nuclei. Images were acquired using an Olympus VS200 fluorescence slide scanner (Olympus, Tokyo, Japan). Quantitative analysis was performed in ImageJ (v1.53) by assessors who were blinded to the experimental groups. The following metrics were quantified: the GFAP-positive area percentage, the percentage of OLIG2^+^ cells relative to DAPI^+^ cells, the percentage of NeuN^+^ cells relative to DAPI^+^ cells, the NF200^+^ area ratio (%), the 5-HT3A receptor-positive area percentage, and, only in the cell transplantation groups, the percentage of NeuN^+^/GFP^+^ double-positive cells.

### 2.15 Statistical analysis

All quantitative data are presented as the mean ± standard deviation (SD). Statistical analyses were performed using GraphPad Prism (Version 10, GraphPad Software, San Diego, CA, USA). For comparisons between two groups, an unpaired Student's t-test was applied. For comparisons across three or more groups based on a single variable, a one-way analysis of variance (ANOVA) was used, followed by the Holm-Sidak post hoc test for multiple comparisons. Data sets with two independent variables (e.g., treatment group and time) were analyzed using a two-way ANOVA, followed by an appropriate post hoc test. A p-value of less than 0.05 was considered statistically significant.

## 3 Results

### 3.1 Effects of melatonin on NSCs differentiation and viability

**Fig. 1:**
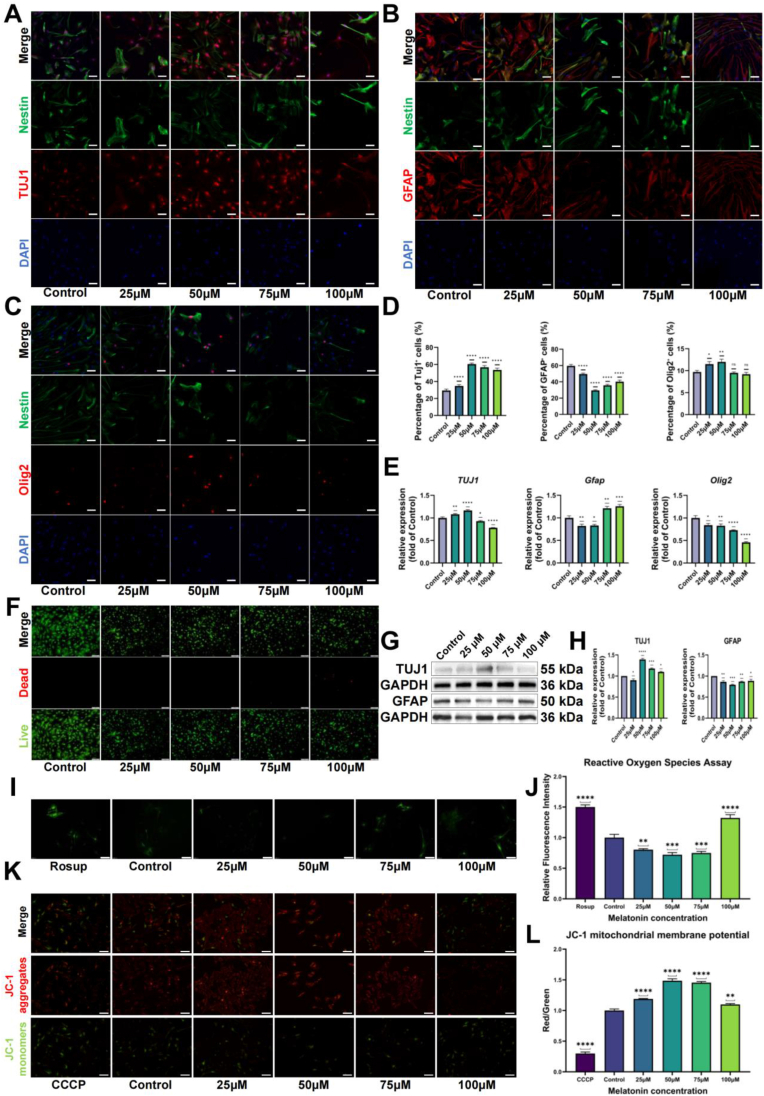
Melatonin dose modulates NSCs lineage commitment, viability, oxidative stress, and mitochondrial membrane potential at day 5. (A) Representative immunofluorescence images of TUJ1 with Nestin in NSCs cultured within the cell-laden MT/BEM matrix and exposed to melatonin (0, 25, 50, 75, 100 μM) for 5 days (scale bar, 50 μm). (B) Representative images of GFAP with Nestin under the same conditions (scale bar, 50 μm). (C) Representative images of Olig2 with Nestin (scale bar, 50 μm). (D) Percentages of TUJ1(+), GFAP (+), and Olig2(+) cells relative to total nuclei (DAPI) (n = 10 fields/group). (E) RT-qPCR of TUJ1, GFAP, and Olig2 normalized to GAPDH and expressed as fold change versus control (ΔΔCt) (n = 6). (F) Live/Dead staining (Calcein AM/EthD-1) at day 5. (G) Western blots of TUJ1 and GFAP with GAPDH loading control. (H) Densitometry of TUJ1/GAPDH and GFAP/GAPDH (n = 3 independent experiments). (I) ROS staining by DCFH-DA with Rosup as the positive control. (J) Quantification of ROS fluorescence intensity (n = 10 fields/group, compared to control). (K) JC-1 staining of mitochondrial membrane potential (ΔΨm) with CCCP as the positive control for depolarization. (L) JC-1 red/green ratio (n = 10 fields/group, compared to control). Statistical analysis: Data are presented as mean ± SD. one-way ANOVA with Holm–Sidak's multiple comparisons for multi-group datasets (D, E, J, L); unpaired two-tailed *t*-test for the two-group comparison (H). Significance: ∗p < 0.05, ∗∗p < 0.01, ∗∗∗p < 0.001, ∗∗∗∗p < 0.0001.

In the standard culture, melatonin demonstrated a potent, dose-dependent effect on NSCs' fate, strongly favoring neuronal differentiation (Fig. 1A–H). As the concentration was increased up to 100 μM, the proportion of TUJ1^+^ neurons rose from 29.40 ± 1.65 cells in the control group to a peak of 60.40 ± 1.27 cells at 50 μM. In contrast, the GFAP^+^ astrocyte population decreased from 59.50 ± 1.84 (control) to 29.50 ± 0.71 cells at 50 μM, while Olig2^+^ cells showed a modest but significant increase at 25 and 50 μM. This pro-neuronal shift was confirmed at the molecular level: at 50 μM, TUJ1 gene expression was upregulated while GFAP and Olig2 expression was down-regulated. Western blotting showed a corresponding increase in TUJ1 protein (1.40-fold) and a decrease in GFAP protein (0.80-fold of control) at the 50 μM concentration (Fig. 1E–G-H). Importantly, this dose window (25-75 μM) showed non-toxic as assessed by the Live/Dead assay (Fig. 1F). Based on these results, which balance maximal astrocyte suppression with robust neurogenesis, a 50 μM concentration was selected for all subsequent experiments.

### 3.2 Reactive oxygen species and mitochondrial function in melatonin-treated NSCs

Consistent with its pro-neuronal effects, melatonin also reduced intracellular ROS and enhanced mitochondrial membrane potential (ΔΨm) within a specific dose range (Fig. 1I–L). Intracellular ROS levels, measured via DCFH-DA fluorescence, dropped to a minimum at 50 μM (0.72-fold vs. control) and remained suppressed at 75 μM, but rebounded past baseline at 100 μM (1.32-fold). Similarly, the JC-1 red/green ratio, a direct measure of Δψm, peaking at 50 μM (1.49-fold vs. control) and remaining elevated at 75 μM before declining toward baseline at 100 μM. The positive (Rosup) controls yielded the expected results. ATP levels, measured using a luciferase-based assay, showed similar trends, with a peak at 50 μM (1.66-fold vs. control) and a decline at higher concentrations, paralleling the changes observed in mitochondrial function (Fig. S2A). Together, these data showed that melatonin most effectively attenuated oxidative stress and improved mitochondrial function within the 25-75 μM range, further supporting 50 μM as the optimal dose for subsequent experiments.

### 3.3 RNA sequencing and functional enrichment of melatonin-treated NSCs

**Fig. 2:**
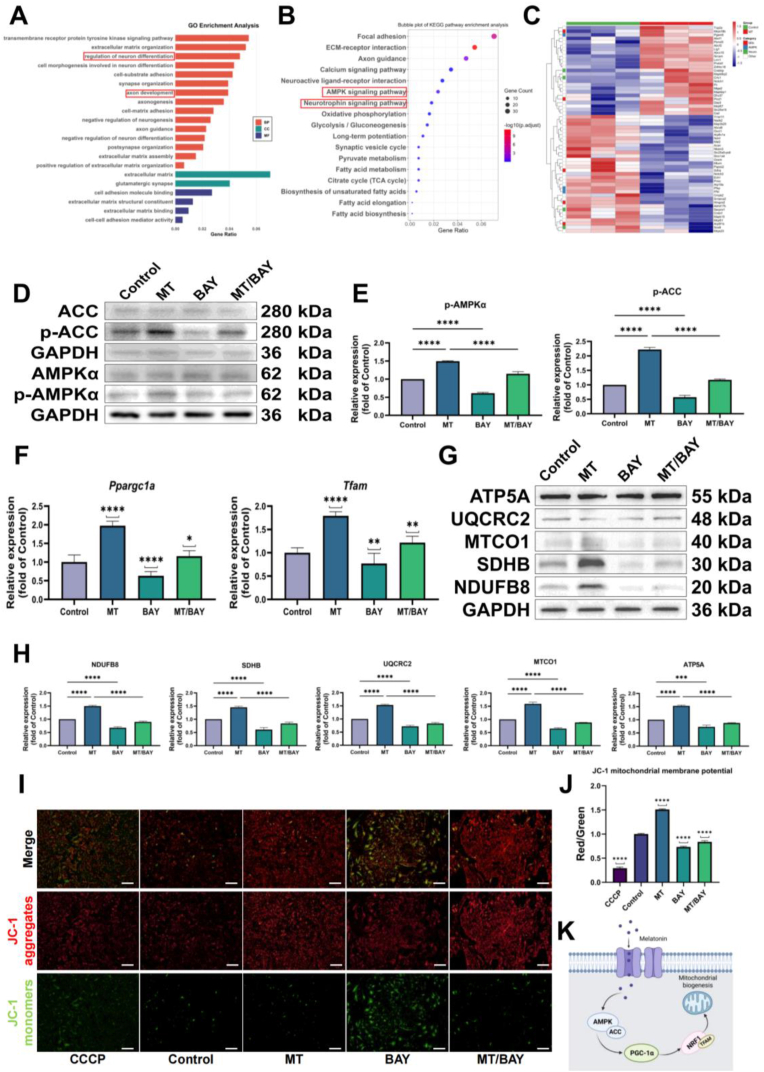
Melatonin activates AMPK signaling and enhances mitochondrial function in vitro. (A) GO enrichment bar plot of differentially expressed genes (DEGs) between Control and Melatonin-treated NSCs. (B) KEGG pathway enrichment bar plot of DEGs between Control and Melatonin groups. (C) Heatmap of selected DEGs associated with neuronal differentiation and mitochondrial function. DEGs were defined as transcripts with FDR <0.05. (D) Representative Western blots showing phosphorylated AMPK (p-AMPK, Thr172) and phosphorylated ACC (p-ACC, Ser79) in Control, Melatonin, Inhibitor, and Melatonin + Inhibitor groups. (E) Densitometric analysis of p-AMPK/total AMPK and p-ACC/GAPDH ratios. (F) RT-qPCR analysis of Ppargc1a and Tfam expression, normalized to GAPDH and presented as fold change relative to the Control group. (G) Representative Western blots of mitochondrial oxidative phosphorylation (OXPHOS) complexes I-V. (H) Densitometric quantification of OXPHOS complexes I-V, normalized to GAPDH (or the corresponding loading control). (I) Representative JC-1 fluorescence images indicating mitochondrial membrane potential (ΔΨm). (J) Quantification of the red/green JC-1 fluorescence ratio from (I). (K) Schematic representation of the proposed melatonin-AMPK-ACC-PGC-1α-NRF1/TFAM signaling axis driving mitochondrial biogenesis in NSCs. All quantitative data (E, F, H, J) are presented as mean ± SD. Statistical significance was assessed using one-way ANOVA followed by Holm–Sidak's multiple comparisons test. ∗p < 0.05, ∗∗p < 0.01, ∗∗∗p < 0.001, ∗∗∗∗p < 0.0001. Fig. 2K created with BioRender.com.

Bulk RNA sequencing of NSCs differentiation with melatonin (50 μM) versus a solvent control revealed transcriptomic shifts consistent with a neurogenic bias and enhanced mitochondrial metabolism (Fig. 2A–C). Differential gene expression analysis between MT and control groups was visualized using a volcano plot (Fig. S3), which highlighted the upregulated (red) and downregulated (green) genes based on statistical significance (p-value ≤0.05) and fold change (|log2 fold change| ≥1). GO enrichment analysis of upregulated genes highlighted terms related to neuron differentiation, axon development, and synapse organization. KEGG pathway analysis was concordant, showing strong enrichment for neurotrophin and AMPK signaling, as well as the key metabolic pathways such as oxidative phosphorylation, the citrate (TCA) cycle, and fatty acid metabolism. GSEA enrichment plots further emphasize the significant enrichment of mitochondrial and neuronal pathways in MT-treated neural stem cells, indicating enhanced mitochondrial function and neuronal differentiation (Fig. S4). The heatmap of representative genes from these categories provides a visual summary of these coordinated transcriptional changes. A concentric PPI network further illustrates that MT treatment coordinately reshapes signaling, metabolic/mitochondrial pathways, and ECM-cell-cycle regulators in NSCs (Fig. S5).

### 3.4 Validation of signaling and metabolic adaptations in vitro

To elucidate the molecular mechanisms underlying melatonin's pro-neuronal effects on NSCs, we first investigated activation of the AMPK signaling pathway. Western blot analysis revealed that melatonin treatment (MT group) significantly increased the phosphorylation levels of both AMPK (1.496 ± 0.012-fold) and ACC (2.220 ± 0.076-fold), compared to control. In contrast, treatment with an AMPK inhibitor (BAY group) suppressed the basal phosphorylation of both AMPK and ACC. Importantly, the melatonin-induced increase in phosphorylation was significantly attenuated when co-administered with the inhibitor (MT/BAY group), confirming that melatonin exerts its effects through the AMPK pathway (Fig. 2D and E). Given that AMPK is a master regulator of mitochondrial biogenesis, we next assessed whether its activation led to changes in mitochondrial function. As expected, melatonin treatment led to a significant upregulation in the protein levels of all five representative oxidative phosphorylation (OXPHOS) complex subunits: NDUFB8 (Complex I, 1.497 ± 0.033), SDHB (Complex II, 1.456 ± 0.045), UQCRC2 (Complex III, 1.532 ± 0.027), MT-CO1 (Complex IV, 1.592 ± 0.060), and ATP5A (Complex V, 1.528 ± 0.032) relative to the control. This broad enhancement of mitochondrial protein expression was essentially reversed by the AMPK inhibitor, indicating that melatonin promotes mitochondrial protein expression via AMPK signaling (Fig. 2G and H).

To determine that a coordinated transcriptional program drove this protein upregulation, we analyzed the expression of key regulatory genes. qPCR results showed that melatonin significantly upregulated the mRNA expression of the master metabolic regulator Ppargc1a (1.976 ± 0.120-fold) and the essential mitochondrial transcription factor Tfam (1.792 ± 0.087-fold). This transcriptional activation also dependent on AMPK activity, as it was blocked by the inhibitor (Fig. 2F). Consistent with these findings, a comprehensive analysis revealed similar AMPK-dependent regulatory patterns in other genes related to the AMPK pathway, mitochondrial biogenesis, and metabolic switching, as shown in detail in Fig. S6.

Ultimately, we sought to determine if these molecular and protein-level changes translated into tangible improvements in mitochondrial function. Mitochondrial activity was assessed using a fluorescent probe, revealing a significantly enhanced fluorescence signal in melatonin-treated NSCs, which was indicative of the improved mitochondrial functional integrity. Quantitative analysis confirmed a substantial increase in mitochondrial function, which was completely abrogated by the AMPK inhibitor (Fig. 2I and J). Similarly, ATP levels showed a trend consistent with the JC-1 results, further supporting the improvement in mitochondrial function (Fig. S2B). A schematic of the proposed melatonin-AMPK-ACC-PGC-1α-NRF1/TFAM signaling axis driving mitochondrial biogenesis in NSCs is shown in Fig. 2K.

### 3.5 Characterization of BEM and MT/BEM hydrogels

**Fig. 3:**
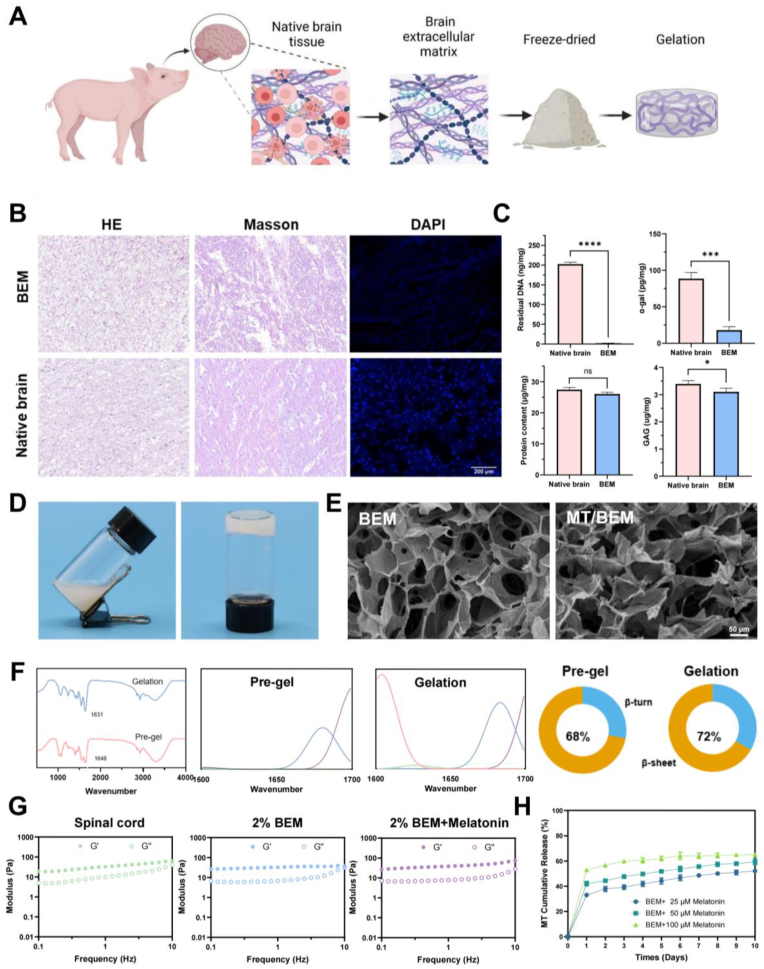
Physicochemical and histological characterization of BEM and MT/BEM hydrogels. (A) Workflow of brain tissue decellularization and preparation of BEM solution for hydrogel formation. (B) Histological analysis of native brain versus BEM after decellularization using H&E, Masson's trichrome, and DAPI staining, confirming the removal of cellular material while preserving matrix architecture (scale bars = 200 μm). (C) Biochemical quantification of residual DNA (ng/mg dry weight), total protein (μg/mg), GAG (μg/mg) and α-gal (pg/mg) content in native brain compared to BEM to assess decellularization efficacy and matrix retention (n = 3 independent preparations). (D) Macroscopic visualization of injectability and the thermally induced sol-gel transition by vial inversion after 10 min at 37 °C. (E) SEM images of freeze-dried BEM and MT/BEM showing interconnected porous architectures (scale bars = 50 μm; representative of n = 3 independent batches). (F) FTIR characterization of the BEM hydrogel sol-gel transition and secondary structure analysis, indicating the gelation process is mediated by the self-assembly of peptides. (G) Small-amplitude oscillatory frequency sweeps at 37 °C showing the comparable storage (G′, filled symbols) and loss (G″, open symbols) moduli for spinal cord tissue, 2% BEM, and 2% BEM + melatonin (n = 3). (H) Cumulative release profile of melatonin in the BEM hydrogel (n = 3), demonstrating a sustained release behavior over 10 days. Fig. 3A created with BioRender.com.

To formulate a bioactive scaffold, a lyophilized BEM powder was produced using a multi-step decellularization workflow (Fig. 3A). Histological analysis confirmed the successful removal of cellular material while preserving the native matrix architecture. In contrast to native brain tissue, the decellularized BEM was devoid of DAPI-positive nuclei yet retained a complex extracellular structure, including collagen networks as confirmed by H&E and Masson's trichrome staining (Fig. 3B). A high decellularization efficiency was confirmed by the residual DNA content less than 1% and immunogenic α-gal to 20%. Conversely, the bioactive components were well preserved: total protein content showed no significant difference compared to native tissue, and glycosaminoglycans (GAG) retention was maintained over 90% (Fig. 3C). The BEM pre-gel solution was readily injectable and underwent a rapid sol-gel transition, forming a stable hydrogel within approximately 10 min at 37 °C (Fig. 3D). To elucidate the mechanism underlying the sol-gel transition of the BEM hydrogel, FTIR spectroscopy was employed to monitor structural evolution (Fig. 3F). A notable redshift of the Amide I peak from 1648^−1^ to 1631^−1^ was observed, accompanied by an increase in β-sheet content from 68% to 72%, indicating a conformational shift from disordered peptides to stable β-sheet fibrils.

Scanning electron microscopy (SEM) revealed that both the BEM and melatonin-loaded BEM (MT/BEM) hydrogels possessed a highly interconnected and porous structure, which permitted the diffusion of nutrients and oxygen (Fig. 3E). Notably, the incorporation of melatonin showed little effect on the porous structure or gelation behavior. These findings indicate that the MT/BEM hydrogel retains the essential physical characteristics of the BEM scaffold, validating its use as an injectable cell delivery vehicle for subsequent studies. Rheological analysis indicated a stable internal network structure in BEM hydrogels across all tested concentrations (1-3 wt%), as evidenced by a storage modulus (G′) higher than the loss modulus (G″) (Fig. S7). Notably, at 1 Hz, the storage modulus of the 2 wt% BEM hydrogel was similar to that of native neural tissue (Fig. 3G). Melatonin loaded into the BEM solution before gelation, exhibited sustained release from the MT/BEM hydrogel. Release profiling showed an initial burst release of melatonin from the BEM hydrogel (Fig. 3H), providing early exposure compatible with the initial differentiation window of transplanted NSCs. Given that this modulus is more conducive to in vivo repair of spinal cord tissue, the 2% BEM concentration was selected for subsequent experiments.

### 3.6 NSCs differentiation in the three-dimensional BEM

**Fig. 4:**
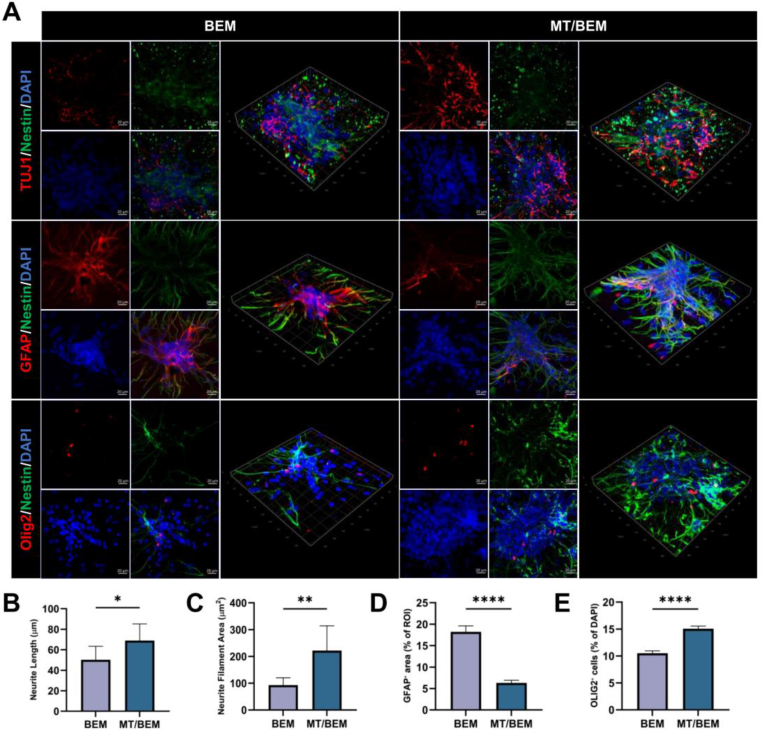
Three-dimensional immunofluorescence and transcriptomic profiling of melatonin-treated NSCs. (A) Representative 3D confocal reconstructions of NSCs networks cultured for 5 days in BEM or MT/BEM hydrogels, immunostained for Nestin, TUJ1, GFAP and OLIG2 with DAPI nuclear counterstain. Scale bar: 50 μm. (B-C) Quantification of neurite outgrowth showing total neurite length (μm) (B) and neurite filament area (μm^2^) (C) per field of view. (D) Quantification of astroglial differentiation expressed as GFAP^+^ area (% of ROI). (E) Quantification of oligodendroglial lineage commitment expressed as OLIG2^+^ cells (% of DAPI^+^ nuclei). Data are presented as mean ± SD. Statistical significance was assessed using an unpaired two-tailed *t*-test; ∗p < 0.05, ∗∗p < 0.01 versus NSCs@BEM.

Before 3D co-culture, the cytocompatibility of BEM with NSCs was evaluated in vitro. The results demonstrate that BEM hydrogels exhibit low cytotoxicity and high biocompatibility toward NSCs, and the corresponding data are provided in the Supplementary Fig. S8. To recapitulate the native physiological niche and assess the potential application of BEM for cell delivery, NSCs were encapsulated within the hydrogel. Confocal microscopy of NSCs cultured for 5 days revealed the 3D organization and differentiation status (Fig. 4A). Maximum-intensity projections of z-stacks and 3D structural views showed that cells in both BEM and MT/BEM formed extensive networks of TUJ1^+^/Nestin^+^ neurites, confirming that BEM supports neuronal differentiation and neurite outgrowth within a 3D matrix. Moreover, consistent with our 2D findings, melatonin incorporation was further promoted neuronal differentiation within the 3D hydrogel. Given the complexity of the dense, overlapping neural networks observed in 3D, the SNT plugin (ImageJ) was used for morphometric quantification in neurite length and neurite filament area, as well as GFAP^+^ area and OLIG2^+^ cell fractions, as well as GFAP^+^ area and OLIG2^+^ cell fractions (Fig. 4B–E). Results revealed a ∼1.5-fold increase in neurite length and a ∼2.4-fold expansion in neurite filament area in the MT/BEM group compared to BEM alone. Quantitative fluorescence analysis also showed a significant reduction in GFAP^+^ astrocytic area accompanied by an increase in the OLIG2^+^ cell fraction, indicating a lineage shift toward oligodendroglial differentiation in the MT/BEM group. Together, these results confirm that melatonin promotes neurite outgrowth and directs lineage commitment toward neuronal and oligodendroglial fates.

### 3.7 Behavioral recovery after SCI

**Fig. 5:**
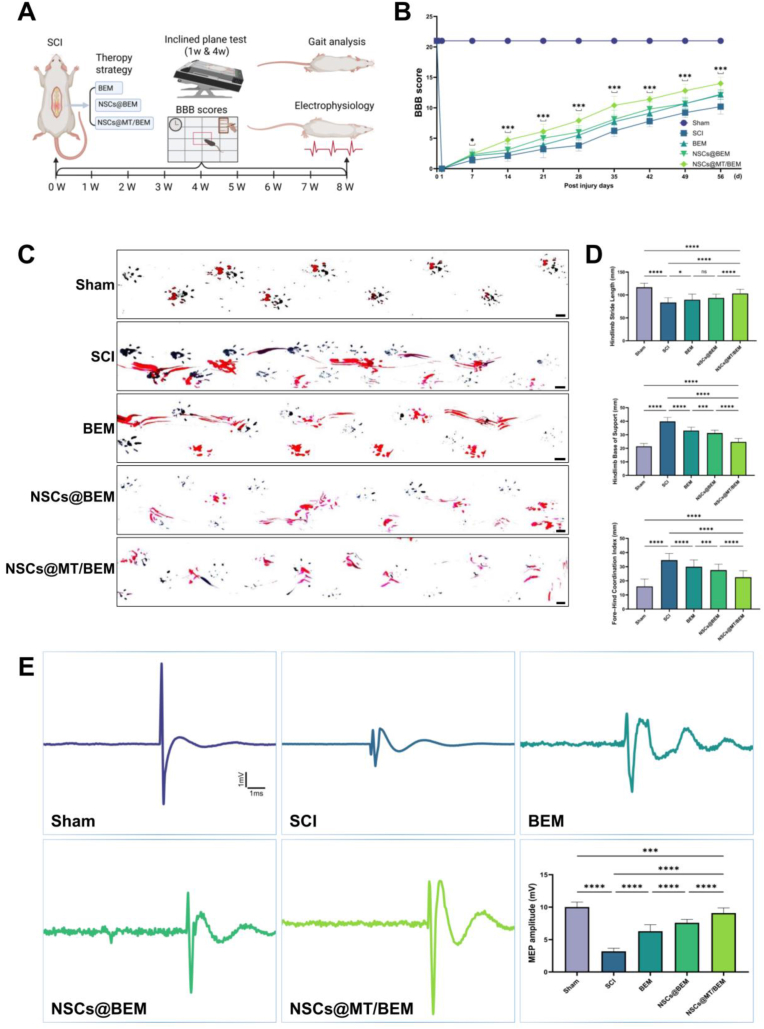
Behavioral and electrophysiological assessments after spinal cord injury. (A) Schematic overview of the SCI model, treatment allocation, and timeline of behavioral and electrophysiological assessments. (B) BBB locomotor scores over time (n = 10 per group). (C) Representative footprint/gait images across experimental groups (Sham, SCI, BEM, NSCs@BEM, NSCs@MT/BEM). (D) Quantitative gait analysis including Hindlimb Stride Length (n = 40), Hindlimb Base of Support (n = 40), and Fore-Hind Coordination Index (n = 100). (E) Representative motor-evoked potential (MEP) waveforms and peak-to-peak amplitude (mV) analysis (n = 15 per group). Statistical analysis: one-way ANOVA with Holm–Sidak's multiple comparisons for endpoint gait metrics (D) and MEP amplitudes (E); All quantitative data are presented as mean ± SD. Two-way repeated-measures ANOVA (group × time) with Holm–Sidak's multiple comparisons for BBB scores (B). Significance: ∗p < 0.05, ∗∗p < 0.01, ∗∗∗p < 0.001, ∗∗∗∗p < 0.0001. Fig. 5A created with BioRender.com.

The experimental workflow and assessment timeline are illustrated in Fig. 5A. Based on continuous longitudinal observations, functional improvements became most pronounced at week 8; therefore, this time point was selected for subsequent analyses. At the 8-week endpoint, the NSCs@MT/BEM group demonstrated the most significant effects on the functional recovery across all assessments (Fig. 5B–E). This was particularly evident in a detailed gait analysis. The hindlimb stride length in the NSCs@MT/BEM group (103.3 ± 9.16 mm) was significantly greater than those in the SCI (83.60 ± 10.47 mm; +23.6%), BEM (89.64 ± 12.44 mm; +15.3%), and NSCs@BEM (93.52 ± 8.40 mm; +10.6%) groups. The hindlimb base of support was significantly narrower for the NSCs@MT/BEM group (24.76 ± 2.60 mm) compared to the SCI (39.87 ± 2.86 mm; −37.9%), BEM (33.12 ± 2.38 mm; −26.8%), and NSCs@BEM (31.29 ± 2.17 mm; −23.4%) groups, closely approaching the Sham baseline (21.46 ± 2.16 mm). Fore-hind coordination was also most effectively restored in the NSCs@MT/BEM group (22.53 ± 4.64 mm), showing most tremendous improvement over the SCI (34.65 ± 4.68 mm), BEM (29.96 ± 4.84 mm), and NSCs@BEM (27.61 ± 4.23 mm) groups. In the open-field BBB test, the NSCs@MT/BEM group also achieved the highest locomotor score at week 8 (14.0 ± 1.05). This was a significant improvement compared to the SCI (10.2 ± 1.23; +37.2%), BEM (12.2 ± 0.79; +14.8%), and NSCs@BEM (12.1 ± 0.74; +14.9%) groups. The maximum inclined-plane angle was measured at multiple time points before and after injury to assess motor function recovery, showing significant improvement in hind limb function (Fig. S9).

These behavioral improvements were supported by electrophysiological improvement of restored neural conduction. The peak-to-peak amplitude of motor-evoked potentials (MEPs) was significantly higher in the NSCs@MT/BEM group (9.087 ± 0.790 mV) compared to both the SCI (3.192 ± 0.469 mV; ∼2.85 × increase) and BEM (6.292 ± 0.999 mV; ∼1.4 × increase) groups. Notably, this level of electrophysiological function was comparable to that of the uninjured Sham group (10.02 ± 0.759 mV). At 8 weeks post-injury, treatment with NSCs@MT/BEM significantly decreased both residual urine volume and manual micturition, suggesting an improvement in bladder function following spinal cord injury (Fig. S10).

### 3.8 Histology and immunofluorescence investigations

**Fig. 6:**
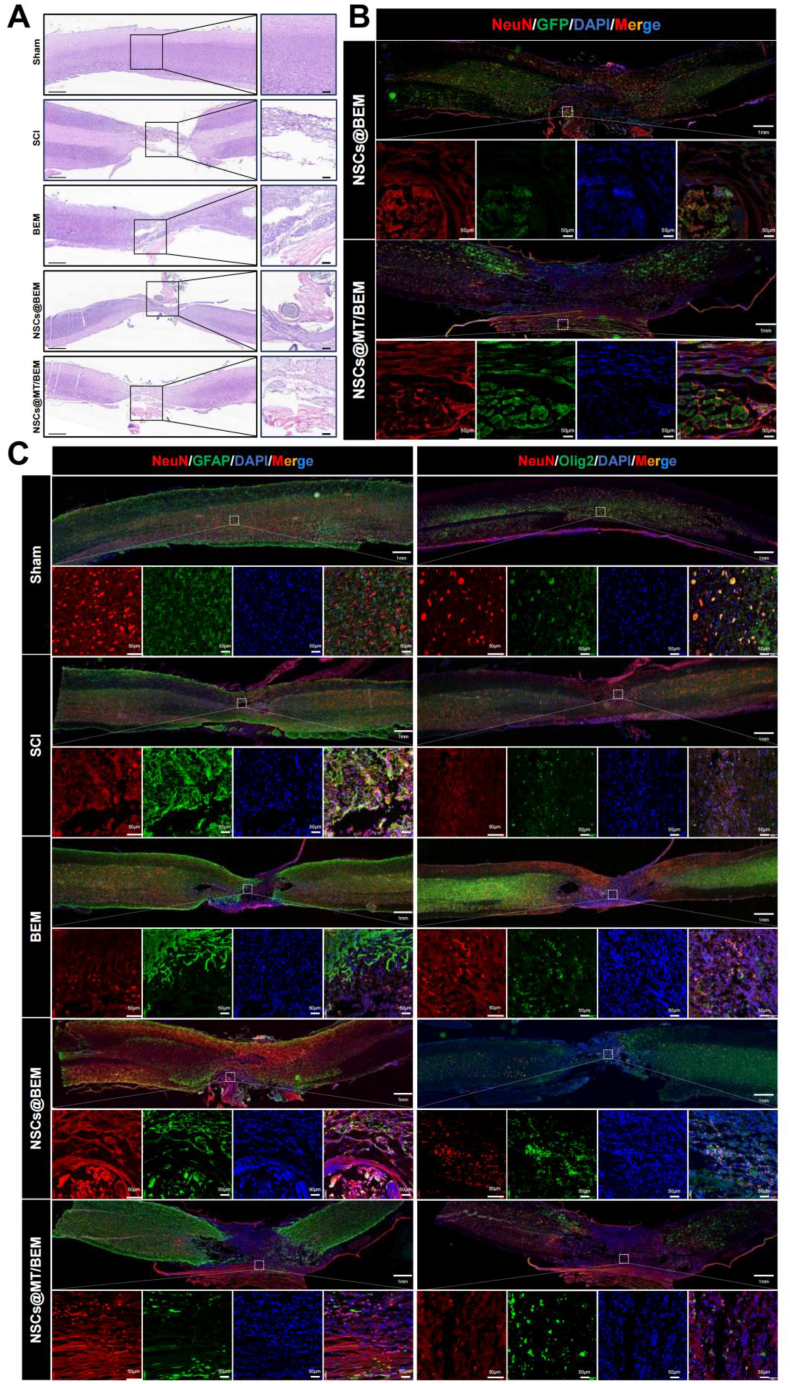
Histology and immunofluorescence of spinal cord repair across treatment groups. Animals were assigned to five groups: Sham, SCI, BEM, NSCs@BEM, and NSCs@MT/BEM. (A) H&E staining of lesion sites (large panels, scale bar 1 mm; insets, 200 μm). (B) Immunofluorescence of NeuN/DAPI/GFP/merge to visualize transplanted NSCs in NSCs@BEM and NSCs@MT/BEM (GFP). (C) Immunofluorescence of NeuN/DAPI/GFAP/merge and NeuN/DAPI/Olig2/merge across groups. For immunofluorescence, large panels use a 1 mm scale bar and insets use 50 μm. Quantitative analyses for neuronal and glial markers appear in Fig. 7.

Histological examination of longitudinal spinal cord sections at 8 weeks post-injury revealed distinct differences in tissue architecture across the groups (Fig. 6A). While H&E staining showed a large, persistent lesion cavity in the SCI group, both the NSCs@BEM and NSCs@MT/BEM groups displayed enhanced tissue preservation and cellularity. Notably, the NSCs@MT/BEM group exhibited the most extensive tissue remodeling, suggesting the most effective restoration of the spinal cord's structural integrity.

**Fig. 7:**
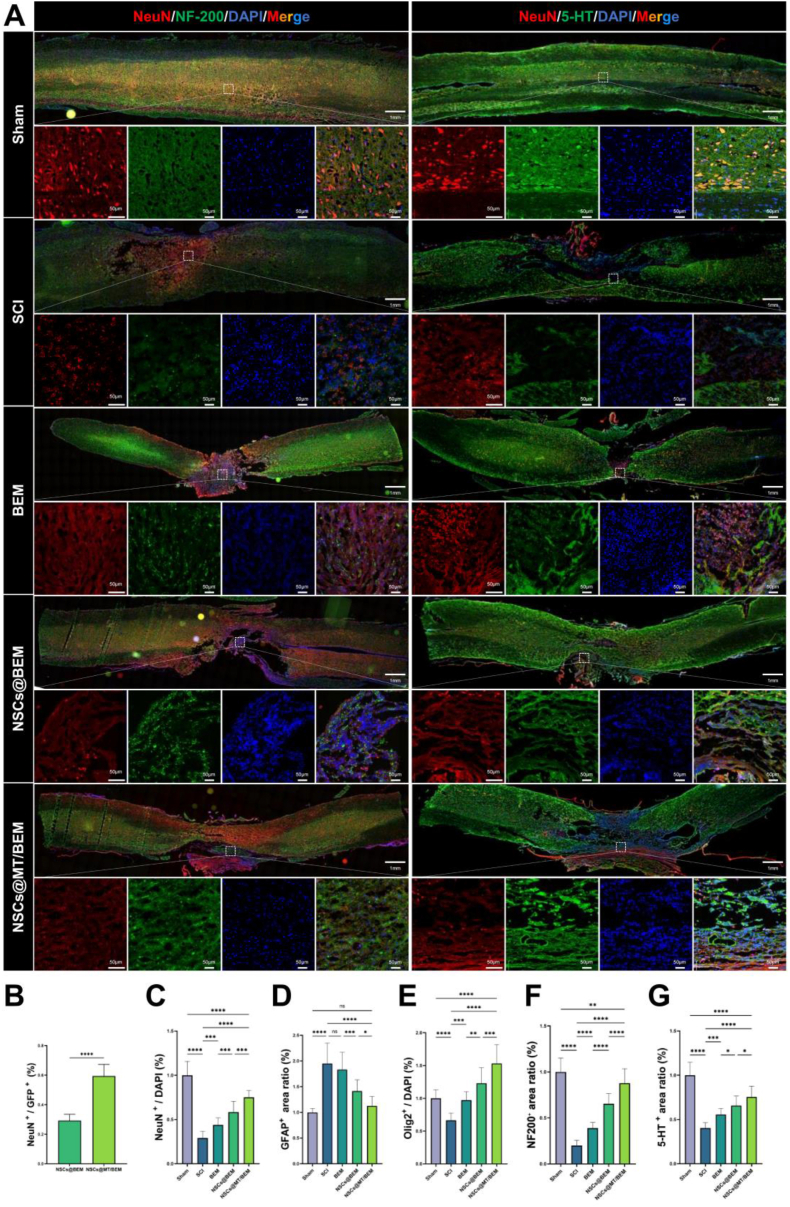
Axonal innervation and quantitative analyses of neuronal/glial outcomes. (A) Representative immunofluorescence images of NeuN/DAPI/5-HT/merge and NeuN/DAPI/NF200/merge across groups (Sham, SCI, BEM, NSCs@BEM, NSCs@MT/BEM) (overview scale bar, 1 mm; inset, 50 μm). (B-G) Quantification of GFP co-localized neuronal ratio (B), NeuN^+^/DAPI^+^ (%) (C), GFAP^+^ area ratio (%) (D), Olig2^+^/DAPI^+^ (%) (E), 5-HT^+^ area ratio (%) (F), and NF200^+^ area ratio (%) (G) (n = 15/group; analysis performed in a blinded manner). All quantitative data are presented as mean ± SD. One-way ANOVA with Holm–Sidak's multiple comparisons test. Significance: ∗p < 0.05, ∗∗p < 0.01, ∗∗∗p < 0.001, ∗∗∗∗p < 0.0001.

To characterize the cellular events underlying this repair, we performed detailed immunofluorescence analysis (Fig. 6B and C; Fig. 7A). First, we assessed the survival of transplanted NSCs. Although successful engraftment was confirmed in both cell therapy groups, quantitative analysis revealed a significantly higher percentage of GFP^+^ cells in the NSCs@MT/BEM group (0.59 ± 0.08) compared to the NSCs@BEM group (0.29 ± 0.04), indicating enhanced NSC survival with melatonin (Fig. 7B). This improved survival was associated with greater neuroprotection. The NSCs@MT/BEM group demonstrated the highest level of NeuN expression (0.75 ± 0.08), significantly reducing the neuronal loss seen in the SCI (0.23 ± 0.08) and BEM (0.44 ± 0.08) groups, and also surpassing the NSCs@BEM group (0.58 ± 0.12) (Fig. 7C).

The combination therapy also significantly modulated the glial response. Reactive astrogliosis, measured by GFAP intensity, was markedly increased in the SCI group (1.95 ± 0.39). The NSCs@MT/BEM group (1.13 ± 0.18) showed the greatest reduction in GFAP intensity, with GFAP levels substantially lower than those in the NSCs@BEM (1.41 ± 0.22) and BEM (1.83 ± 0.34) groups (Fig. 7D). Conversely, the treatment promoted the expansion of the oligodendrocyte lineage. The number of Olig2-positive cells was highest in the NSCs@MT/BEM group (1.53 ± 0.29), which was significantly greater than in the NSCs@BEM (1.23 ± 0.24), BEM (0.97 ± 0.13), and SCI (0.66 ± 0.11) groups (Fig. 7E).

Finally, we evaluated axonal regeneration using both NF200 and 5-HT immunostaining. The NF200^+^ area ratio was profoundly reduced in the SCI group (0.20 ± 0.06), indicating extensive axonal disruption. Partial recovery was observed in the BEM (0.39 ± 0.06) and NSCs@BEM (0.65 ± 0.11) groups, whereas the NSCs@MT/BEM group exhibited the most substantial restoration of axonal profiles (0.88 ± 0.16), approaching the level observed in the Sham group (1.00 ± 0.15) (Fig. 7F). Consistently, the density of 5-HT^+^ serotonergic fibers, severely diminished in the SCI group (0.40 ± 0.06), was most robustly increased in the NSCs@MT/BEM group (0.75 ± 0.12). This represented a significant improvement over both the BEM (0.56 ± 0.07) and NSCs@BEM (0.66 ± 0.11) groups, indicating enhanced long-tract axonal regeneration in the NSCs@MT/BEM group (Fig. 7G). Additional exploratory immunofluorescence observations in the NSCs@MT/BEM group at 10 weeks post-injury further showed persistence of graft-derived GFP^+^ cells, NeuN^+^ neurons, reduced GFAP immunoreactivity, and maintained NF200/5-HT-positive axonal profiles, consistent with a sustained pro-regenerative tissue phenotype (Fig. S11).

### 3.9 Molecular validation in SCI repair

**Fig. 8:**
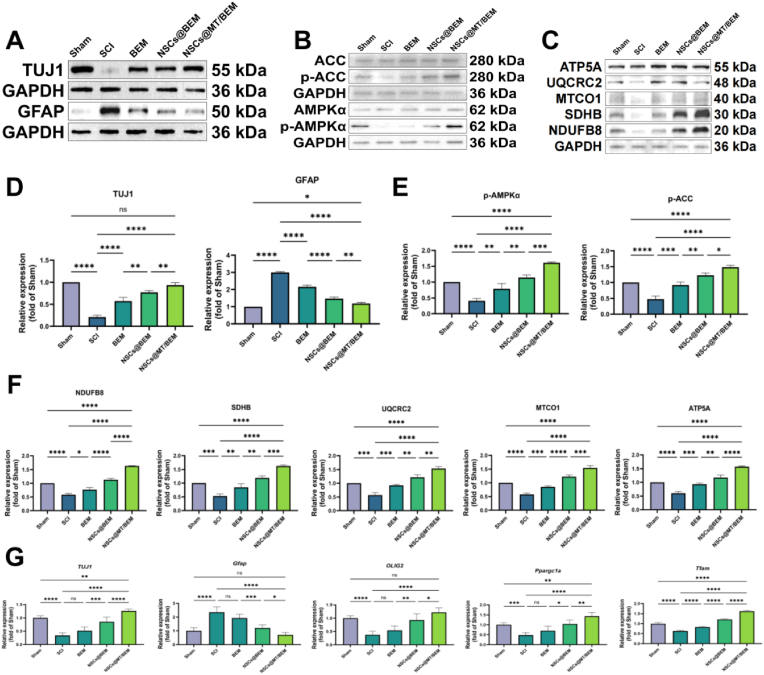
Molecular validation of neural repair and mechanism activation in spinal cord tissue. Western blot and qPCR analyses of spinal cord tissue lysates from Sham, SCI, BEM, NSCs@BEM, and NSCs@MT/BEM groups. (A) Representative Western blots for the neuronal marker TUJ1 and the glial scar marker GFAP. (B) Representative Western blots for phosphorylated AMPK (p-AMPK), phosphorylated ACC (p-ACC), and their respective total proteins. (C) Representative Western blots for the five oxidative phosphorylation (OXPHOS) complex subunits. (D) Densitometric quantification of TUJ1 and GFAP protein levels. (E) Densitometric quantification of the p-AMPK/total AMPK and p-ACC/total ACC ratios. (F) Densitometric quantification of OXPHOS complex protein levels. (G) Relative mRNA expression of neural markers (TUJ1, GFAP, Olig2) and key mitochondrial biogenesis regulators (Ppargc1a, Tfam) determined by qPCR. Data are presented as mean ± SD. Statistical significance was determined by one-way ANOVA with Holm–Sidak's multiple comparisons test. (∗p < 0.05, ∗∗p < 0.01, ∗∗∗p < 0.001, ∗∗∗∗p < 0.0001).

To validate in vitro findings in the animal model and assess the molecular underpinnings of functional recovery, we performed molecular analyses on spinal cord tissue lysates 28 days post-injury. At the protein level, Western blot analysis confirmed the treatment's efficacy on tissue repair. SCI dramatically reduced the neuronal marker TUJ1 (to 0.21 ± 0.05 of Sham levels) while upregulating GFAP (to 3.00 ± 0.05-fold), effects which were most effectively reversed by the NSCs@MT/BEM group, showing the greatest recovery of TUJ1 (to 0.93 ± 0.06) and the most substantial attenuation of GFAP (to 1.19 ± 0.06) (Fig. 8A–D). Mechanistically, these therapeutic effects were linked to the activation of the proposed signaling pathway. The injury-induced suppression of phosphorylated AMPK and ACC, along with diminished expression of all five OXPHOS complex subunits, was robustly reversed by the NSCs@MT/BEM treatment, elevating p-AMPK and p-ACC to levels substantially above those in the Sham group and indicating significant pathway activation and mitochondrial enhancement within the repaired tissue (Fig. 8B, C, E, F).

These protein-level observations were further supported at the transcriptional level by qPCR analysis (Fig. 8G). The mRNA expression of neuronal (TUJ1) and oligodendrocyte (OLIG2) markers, which were downregulated after SCI, was restored by the NSCs@MT/BEM treatment to supra-normal levels (1.26 ± 0.08 and 1.22 ± 0.16, respectively). Concurrently, the injury-induced upregulation of GFAP mRNA was strongly suppressed to a level below that of the Sham group (0.71 ± 0.18). Furthermore, consistent with the mechanistic findings, the expression of key mitochondrial biogenesis regulators, Pgc-1α (1.43 ± 0.18) and Tfam (1.62 ± 0.04), was potently restored by NSCs@MT/BEM treatment. The transcriptional restoration of other downstream mitochondrial component genes is detailed in Supplementary Fig. S12. Consistent with the restoration of AMPK signaling and OXPHOS proteins, ATP content in lesion-segment spinal cord tissue at 4 weeks post-injury was increased in the treatment groups, with the highest level in the NSCs@MT/BEM group (Fig. S13). Collectively, these in vivo molecular data support that the NSCs@MT/BEM treatment promotes neural repair and inhibits gliosis by activating the AMPK-PGC-1α signaling axis and upregulating mitochondrial biogenesis regulators and OXPHOS complex proteins within the injured spinal cord.

## 4 Discussion

SCI is caused by an instantaneous mechanical injury, followed by a secondary cascade characterized by inflammatory storm, oxidative stress, excitotoxicity, blood-spinal cord barrier disruption, demyelination, and glial scar formation [20]. In the acute phase (<48 h) and sub-acute phase (2 days-2 weeks), a microenvironmental imbalance appears, which is rich in inhibitory molecules (such as CSPGs, Nogo, etc.) and excessive ROS. This leads to obstructed axonal regeneration, broken synaptic circuits, and causes endogenous neural stem cells (eNSCs) to tend towards gliosis rather than neuronal differentiation [[21], [22], [23]]. To promote neuroregeneration after SCI, numerous studies have been conducted, including gene editing, drug intervention, stem cell transplantation, and exosome delivery. However, due to the complex SCI microenvironment, single-drug or simple cell transplantation often fails to achieve lasting recovery. Currently, there is no effective therapy, and multimodal intervention (structural + immune + metabolic) is gradually becoming a mainstream strategy [24,25].

Structural scaffolds, immune regulation, and metabolic reprogramming require synergistic intervention. A key advantage of dECM scaffolds is their ability to preserve the native microenvironment: the multi-component matrix, abundant integrin recognition motifs, GAG-mediated "endogenous reservoir" of growth factors, and a GAG-mediated reservoir of growth factors, together providing a niche that can be "read and reshaped" by cells. The preservation of integrin motifs such as RGD and IKVAV, as well as the GAG-mediated retention of growth factors, are critical for the long-term efficacy of tissue-specific signaling [[26], [27], [28], [29]]. Because the differentiation and maturation of NSCs are highly dependent on tissue-specific cues, porcine brain-derived BEM from neural tissue offers a superior scaffold. BEM is closer to the central nervous tissue in its protein profile and mechanical spectrum, promoting neural lineage growth and axonal elongation [[30], [31], [32]]. Its CNS-specific basement membrane protein profile, high HA/GAG content, and low-modulus, stress-relaxing mechanical spectrum more closely match the in-situ signals of the spinal cord, and can significantly promote NSCs differentiation towards neurons, axonal elongation, and synapse formation, as well as support oligodendrocyte maturation and remyelination. Compared to generic hydrogels such as collagen, alginate, or PEG, BEM achieves multi-level synergistic enhancement across dimensions, including biochemistry, mechanics, immune regulation, and cell-mediated remodeling.

As an endogenous hormone, melatonin offers significant advantages in terms of accessibility and safety due to its biocompatibility and low immunogenicity: its raw materials are widely sourced and low-cost, and its formulations are well established; it can be administered orally and is also convenient for local administration and sustained-release delivery in combination with hydrogels/nanocarriers. Mechanistically, MT exerts a dual antioxidant effect by directly scavenging free radicals and inducing the expression of antioxidant enzymes. It also reduces inflammation, stabilizes mitochondrial function, and attenuates apoptosis and excitotoxicity. In the nervous system, MT is associated with the AMPK-PGC-1α-TFAM axis and the upregulation of ACC Ser79 phosphorylation [33]. Multiple studies have shown that melatonin can promote mitochondrial biogenesis and respiratory chain complex assembly by activating AMPK and upregulating the PGC-1α/NRF1/TFAM axis, manifesting as increases in mtDNA content, mitochondrial membrane potential (Δψm), oxygen consumption rate (OCR), and ATP generation, accompanied by reduced proton leakage and mitochondrial ROS [[34], [35], [36]]. In neural stem cell/neuron models, melatonin has also been shown to regulate mitochondrial dynamics (DRP1-MFN2/OPA1) and PINK1-Parkin-mediated mitophagy, improve calcium homeostasis and presynaptic energy supply, thereby supporting neurogenesis, axonal growth, and synaptic plasticity [37,38]. NSCs not only differentiate into neurons and oligodendrocytes, but can also extend long-distance axons, form functional synapses with the host, and promote remyelination, thereby obtaining a more direct "circuit reconstruction" potential. Some studies, as evidenced by trans-segmental axonal regeneration and corticospinal tract (CST) reconstruction, demonstrate the possibility of circuit-level integration [39,40].

The accompanying BEM hydrogel can provide protein and glycosaminoglycan cues near the central nervous system, supporting NSC survival, neuronal differentiation, and neurite/axonal growth, thereby demonstrating a tissue-specific biomimetic advantage. Based on this, we developed an injectable hydrogel platform (NSCs@MT/BEM) incorporating porcine brain-derived decellularized matrix, loaded with melatonin and embedded with NSCs. This system not only supports the activation and survival of transplanted cells, but also combines multiple functions, including tissue adhesiveness, biocompatibility, degradability, ROS scavenging, and low immunogenicity.

The injectable porcine brain decellularized matrix hydrogel constructed in this study possesses good biocompatibility, and can serve as a "tissue-specific microenvironment" chassis. We co-assembled melatonin (MT) with rat-derived NSCs (NSCs@MT/BEM), utilizing melatonin's dual-track antioxidant and metabolic regulatory properties to reduce ROS load, stabilize mitochondrial membrane potential, and promote OXPHOS enhancement via the AMPK pathway under the background of oxidative stress. Transcriptomics further indicated that melatonin-treated NSCs were enriched in neurogenesis/synaptic assembly and mitochondrial metabolic pathways, with differentially expressed genes enriched in the AMPK metabolic pathway. In the nervous system, enhanced mitochondrial biogenesis and respiratory chain complex assembly bolster the local energy supply and protein translation within axonal growth cones and presynaptic terminals. This metabolic shift is driven by the activation of the AMPK-PGC-1α-NRF1/TFAM axis and upregulated ACC (Ser79) phosphorylation, indicating a metabolic reprogramming from glycolytic bias toward oxidative phosphorylation (OXPHOS). Consequently, this reprogramming underpins the observed concomitant increase in neuronal markers (TUJ1/NeuN) and decrease in GFAP.

Compared with traditional materials or single interventions, this platform exhibits synergistic advantages in maintaining the survival of transplanted NSCs and their neuronal fate commitment. Regenerating 5-HT axons together with increased NeuN-positive neurons are consistent with improved axonal remodeling and synaptic plasticity within the lesion microenvironment, thereby supporting functional recovery. The 5-HT reinnervation correlates with the improvement of gait parameters. At the same time, the reinnervation of 5-HT pathway depends on the distribution and anchoring of mitochondria within the axon; the enrichment of mitochondria near the nodes of Ranvier and synapses can enhance the stability of the excitatory-inhibitory balance and gait rhythm. Functionally, at the 8th week post-injury, gait parameters and BBB scores were significantly improved, and the peak-to-peak amplitude of cortico-spinal MEP conduction increased; both of these are standardized endpoints in rodent SCI research and form a structure-function coupling with the histological indicators. A positive correlation between MEP improvement and increased NeuN/5-HT axon density has also been frequently reported, supporting structure-function consistency [41,42].

Collectively, the present study offers a conceptual and mechanistic advance in SCI repair by demonstrating that the therapeutic efficacy of transplanted NSCs can be substantially augmented through the coordinated modulation of their structural niche, metabolic state, and injury microenvironment. Unlike previous strategies involved of biomaterials, neuroprotective molecules, or stem-cell transplantation in isolation, our work integrates these components into a unified, mechanistically synergistic platform. We show that the CNS-specific biochemical and viscoelastic cues of BEM not only provide a tissue-matched structural scaffold, but also cooperate with melatonin-mediated metabolic reprogramming to fundamentally reshape the bioenergetic profile of NSCs. Activation of the AMPK-PGC-1α-NRF1/TFAM axis drives mitochondrial biogenesis, enhances OXPHOS efficiency, and suppresses ROS accumulation, thereby shifting NSCs toward a high-energy, pro-neuronal state with greater resistance to the hostile SCI microenvironment. This metabolically optimized phenotype enhances the survival of transplanted NSCs, promotes their robust differentiation into neurons and oligodendroglia, and improves their effectiveness in circuit-level repair.

Importantly, this combinatorial strategy is not merely a biological enhancement but represents a potentially translatable therapeutic paradigm. Both BEM hydrogels and melatonin are clinically accessible materials, and the injectable, in situ-gelling format aligns well with intraoperative workflows. The sustained functional improvements observed across neuronal preservation, glial-scar attenuation, long-distance axonal regeneration, and long-term gait recovery collectively underscore the feasibility of a therapeutic strategy that simultaneously targets structural support, immune-metabolic imbalance, and cellular bioenergetics. Together, these findings establish metabolic rejuvenation of grafted stem cells within a CNS-matched matrix niche as a powerful and previously underappreciated avenue to overcome longstanding barriers in SCI repair.

In summary, NSCs@MT/BEM, through the multi-functional synergy of "tissue-specific microenvironment + metabolic reprogramming", constructs a microenvironment conducive to transplanted NSCs, driving circuit reconstruction and promoting sustained functional recovery after SCI. It should be noted that residual DNA/antigenicity and batch-to-batch variation in xenogeneic dECM are key risks for translation and are often quality-controlled through DNA residue, collagen crosslinking degree, and mechanical and proteomic fingerprints [43]. In the future, control over the ectopic migration/overproliferation of NSCs and the batch-to-batch consistency of BEM should be further optimized and strengthened, and the safety and reproducibility of the therapeutic effect should be verified in a longer-term, multi-center framework.

## CRediT authorship contribution statement

**Rushuo Wei:** Writing – original draft, Visualization, Validation, Software, Methodology, Funding acquisition, Formal analysis, Data curation, Conceptualization. **Quanjing Mei:** Writing – review & editing, Visualization, Validation, Software, Project administration, Methodology, Investigation, Funding acquisition, Formal analysis. **Tiangang Zhou:** Writing – review & editing, Supervision, Project administration, Methodology, Investigation, Formal analysis, Conceptualization. **Xiaoqian Zhang:** Visualization, Validation, Methodology, Formal analysis, Data curation. **Weiqiang Liu:** Writing – review & editing, Supervision, Resources, Project administration, Data curation. **Mingdong Yu:** Writing – review & editing, Supervision, Resources, Project administration, Data curation. **Bingwu Wang:** Writing – review & editing, Visualization, Validation, Supervision, Resources, Project administration, Funding acquisition, Data curation. **Hui-Qi Xie:** Writing – review & editing, Supervision, Resources, Project administration, Data curation, Conceptualization. **Ruzhan Yao:** Writing – review & editing, Supervision, Software, Resources, Project administration, Methodology, Funding acquisition, Data curation, Conceptualization.

## Ethics approval and consent to participate

All animal experiments were reviewed and approved by the Experimental Animal Ethics Committee of Shandong Second Medical University (Approval No. 2021SDL430). All procedures were conducted in accordance with relevant national regulations and institutional guidelines for the care and use of laboratory animals and are reported in compliance with the ARRIVE guidelines.

## Declaration of competing interest

The authors declare that they have no known competing financial interests or personal relationships that could have appeared to influence the work reported in this paper.
