# Supplementary material for: Melatonin-incorporated brain extracellular matrix hydrogel enhances NSCs mitochondrial metabolism to promote neuroregeneration via the AMPK-PGC-1α-NRF1/TFAM axis after spinal cord injury
Source: Bioact Mater. 2026 Apr 9;63:373–89. doi: 10.1016/j.bioactmat.2026.04.006 (PMC13091134; doi:10.1016/j.bioactmat.2026.04.006)
Supplement: Multimedia component 1 [file mmc1.docx]

Supplementary material

Melatonin-incorporated brain extracellular matrix hydrogel enhances NSCs mitochondrial metabolism to promote neuroregeneration via the AMPK-PGC-1α-NRF1/TFAM axis after spinal cord injury

Figure S1. Representative immunofluorescence images of cultured NSCs staining for Nestin (green) and DAPI (blue). Scale bar, 50 µm.


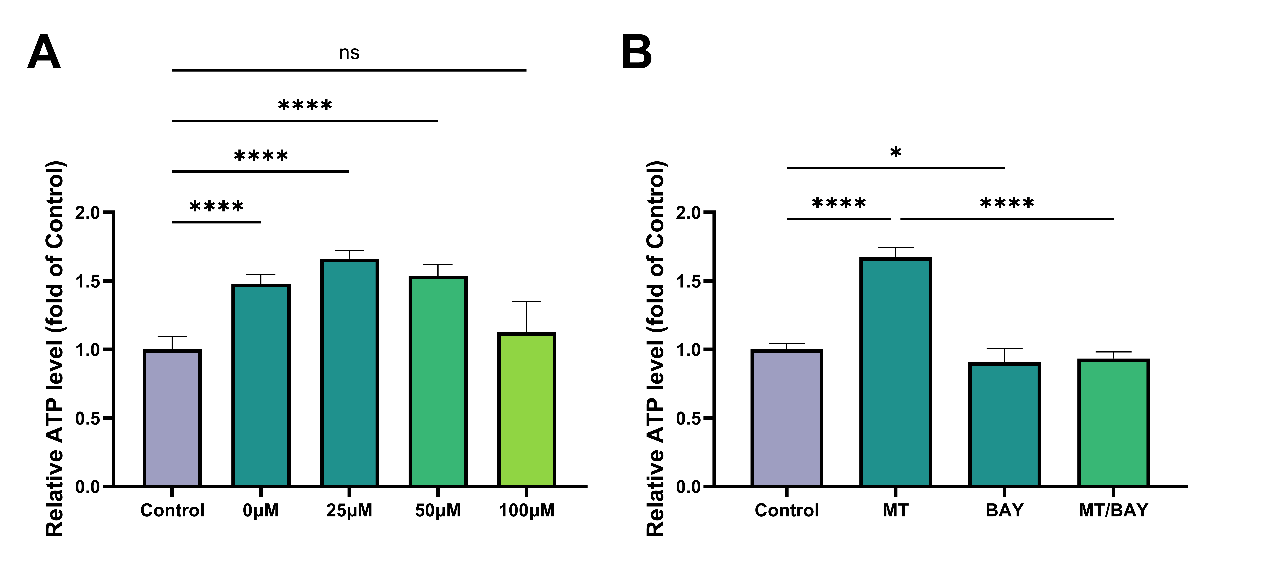


Figure S2. ATP levels in cultured cells. (A) The effect of melatonin on ATP content in neural stem cells across a concentration gradient of 0 to 100 μM. (B) ATP levels in Control, MT, BAY, and MT/BAY groups. ATP content was quantified using a luciferase-based assay (Beyotime, S0026). Values are expressed as fold change relative to the Control. Data are mean ± SD (n = 6/group). One-way ANOVA with Holm–Sidak’s multiple comparisons test; *p < 0.05, ****p < 0.0001.


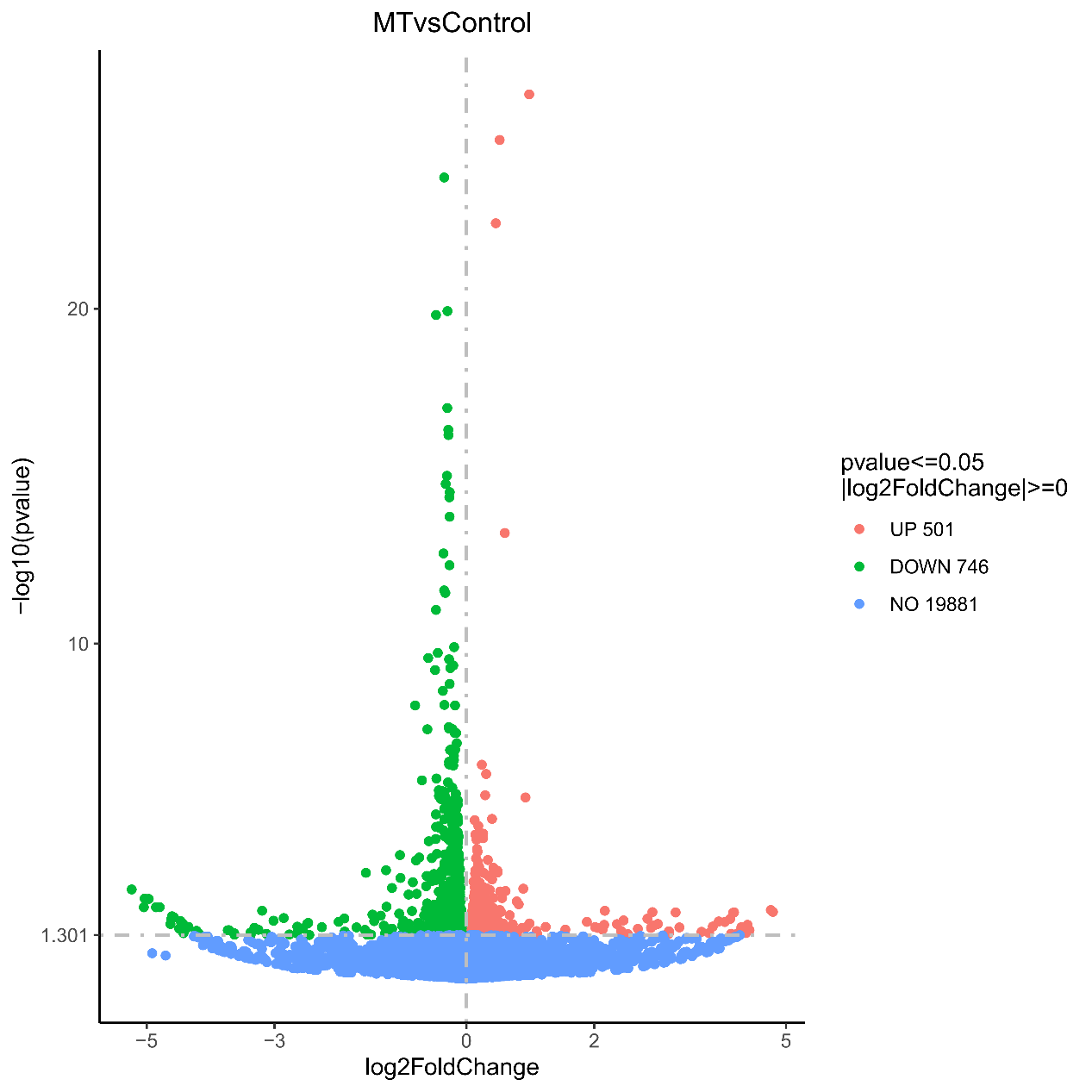


Figure S3. Volcano plot of differential gene expression in MT vs Control. The plot displays the relationship between log2 fold change and -log10(p-value) for genes in the MT vs Control comparison. Red dots represent genes with significant upregulation (p-value ≤ 0.05 and |log2 fold change| ≥ 0), green dots represent genes with significant downregulation, and blue dots represent non-significant genes (no differential expression). A threshold of p-value ≤ 0.05 and |log2 fold change| ≥ 0 was used for significance. The number of upregulated genes is 501, downregulated genes is 746, and non-significant genes is 19881.


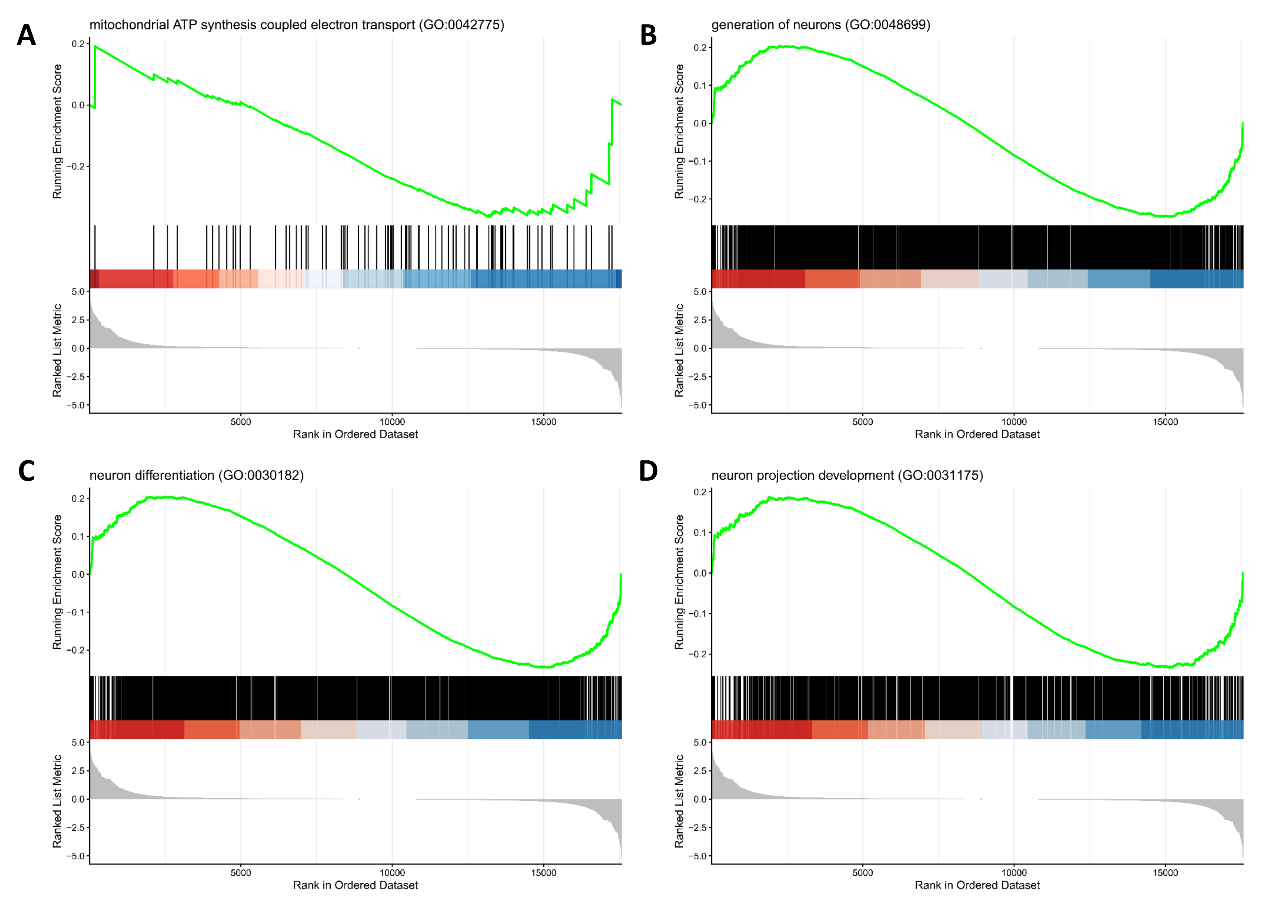


Figure S4. GSEA enrichment plots showing the significant enrichment of mitochondrial and neuronal pathways in MT-treated neural stem cells (NSCs). (A) Mitochondrial ATP synthesis coupled electron transport (GO:0042775), (B) Generation of neurons (GO:0048699), (C) Neuron differentiation (GO:0030182), and (D) Neuron projection development (GO:0031175). The green line represents the running enrichment score (ES), with the x-axis showing the rank in the ordered dataset and the y-axis displaying the ES values. The enrichment of these pathways suggests that MT treatment enhances mitochondrial function and promotes neuronal differentiation and development in NSCs.


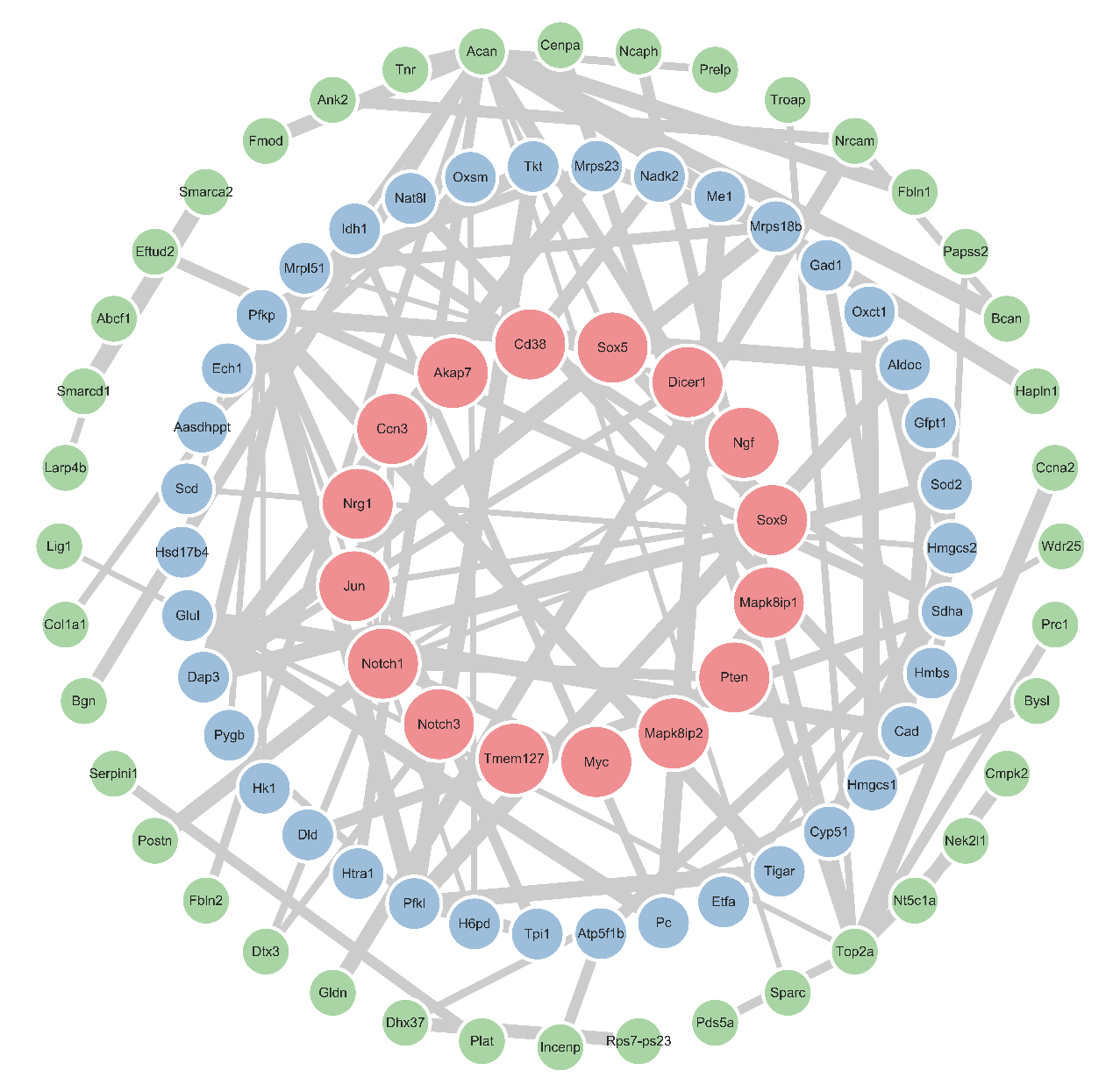


Figure S5. Concentric PPI network of MT-responsive genes in NSCs. Nodes represent proteins and edges denote high-confidence interactions. Nodes are organized into three functional rings: an inner core of signaling and fate regulators (red), a middle metabolic/mitochondrial layer (blue), and an outer layer enriched for ECM/microenvironmental and cell-cycle/chromatin regulators (green).


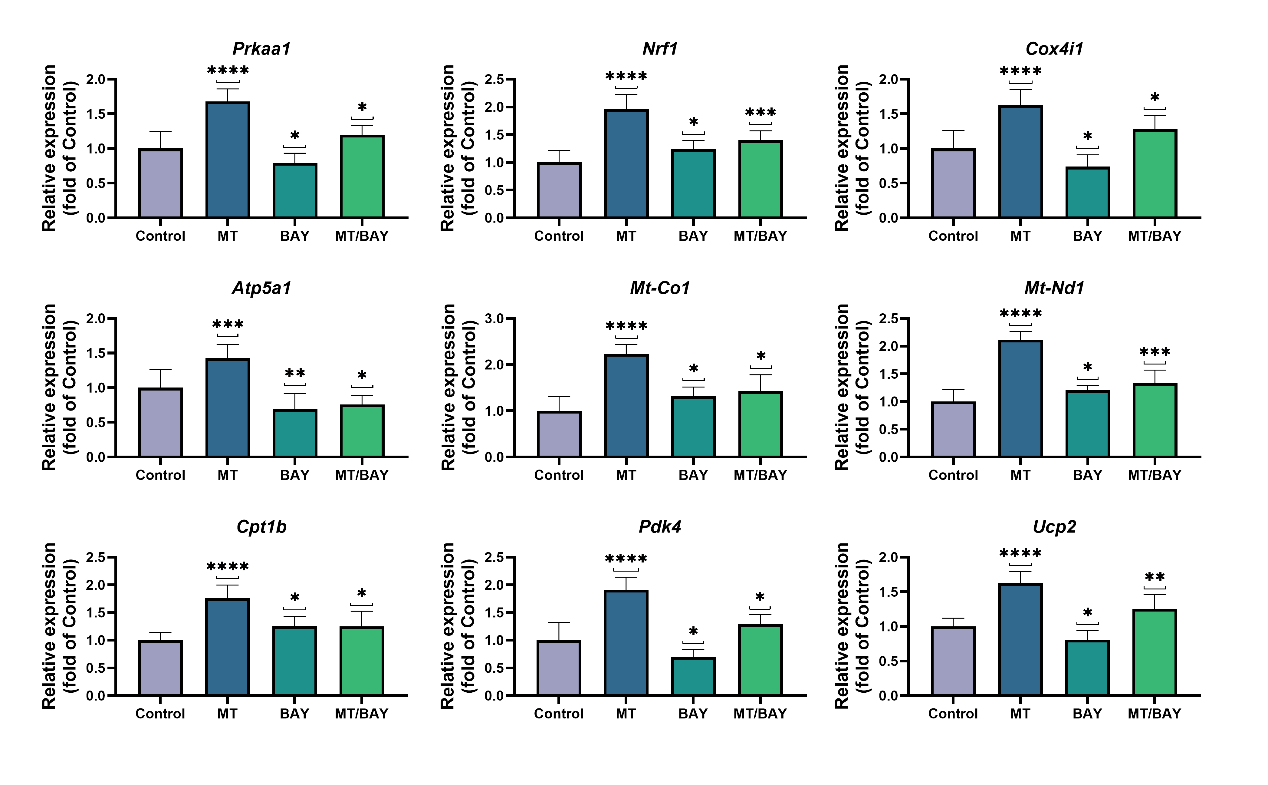


Figure S6. Relative mRNA expression of genes related to the AMPK pathway, mitochondrial biogenesis, and metabolism in NSCs treated with melatonin (MT), an AMPK inhibitor (BAY), or both. *p < 0.05, **p < 0.01, ***p < 0.001, ****p < 0.0001.


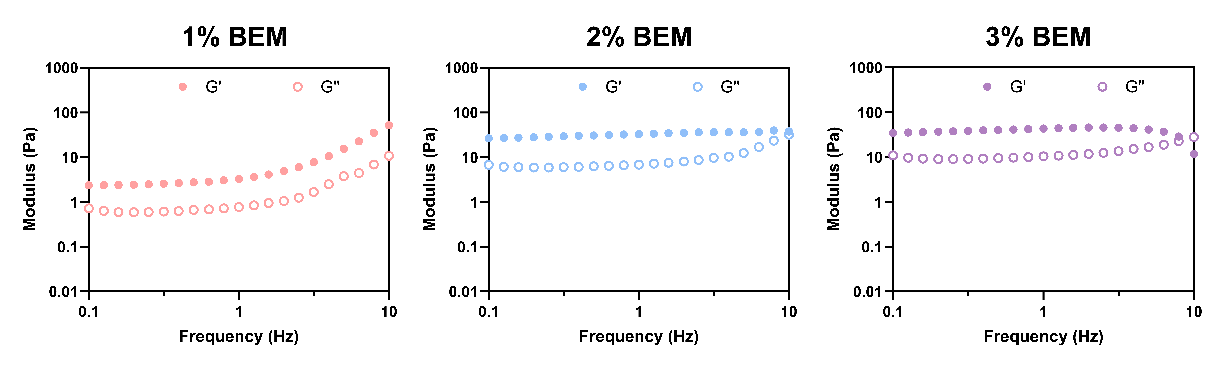


Figure S7. Small-amplitude oscillatory frequency sweeps at 37 °C showing storage (G′, filled symbols) and loss (G″, open symbols) moduli of 1% BEM, 2% BEM, and 3% BEM (n = 3).

Figure S8.
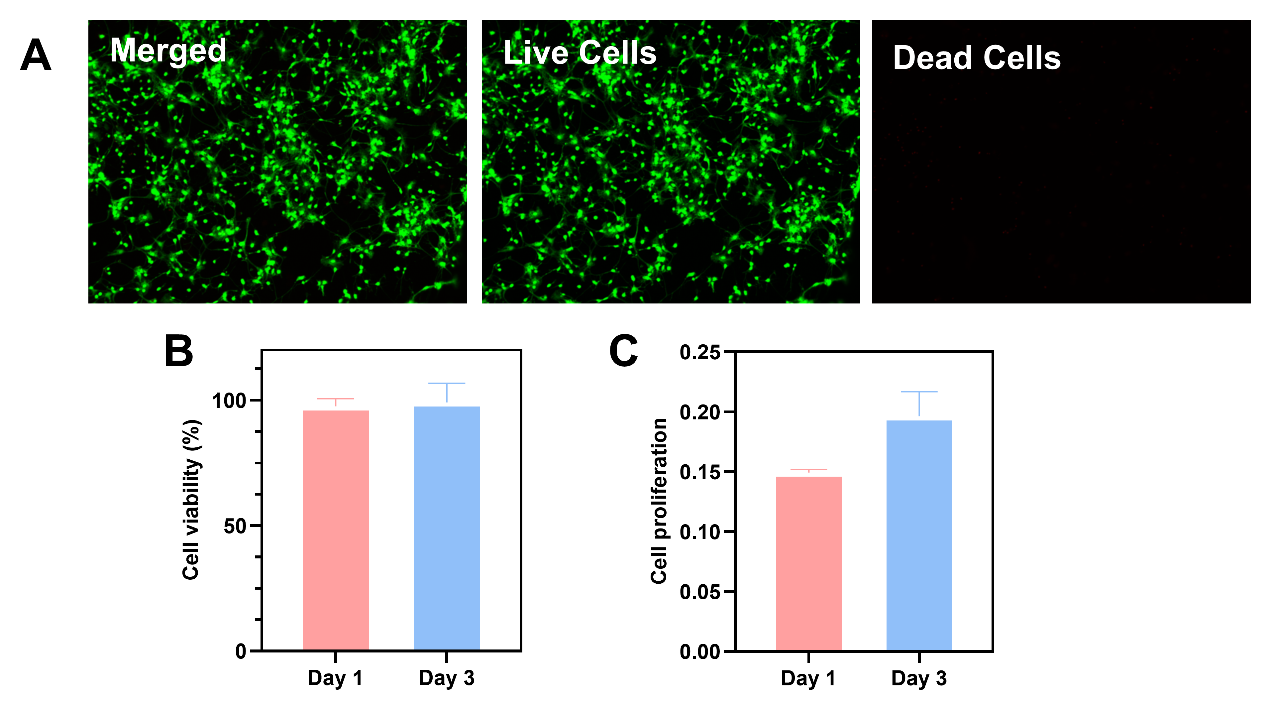
 Cytotoxicity and biocompatibility of BEM. (A) Live/Dead staining of NSCs cultured on BEM at day 3. (B) Quantification of cell viability on days 1 and 3. (C) Cell proliferation of NSCs on days 1 and 3.


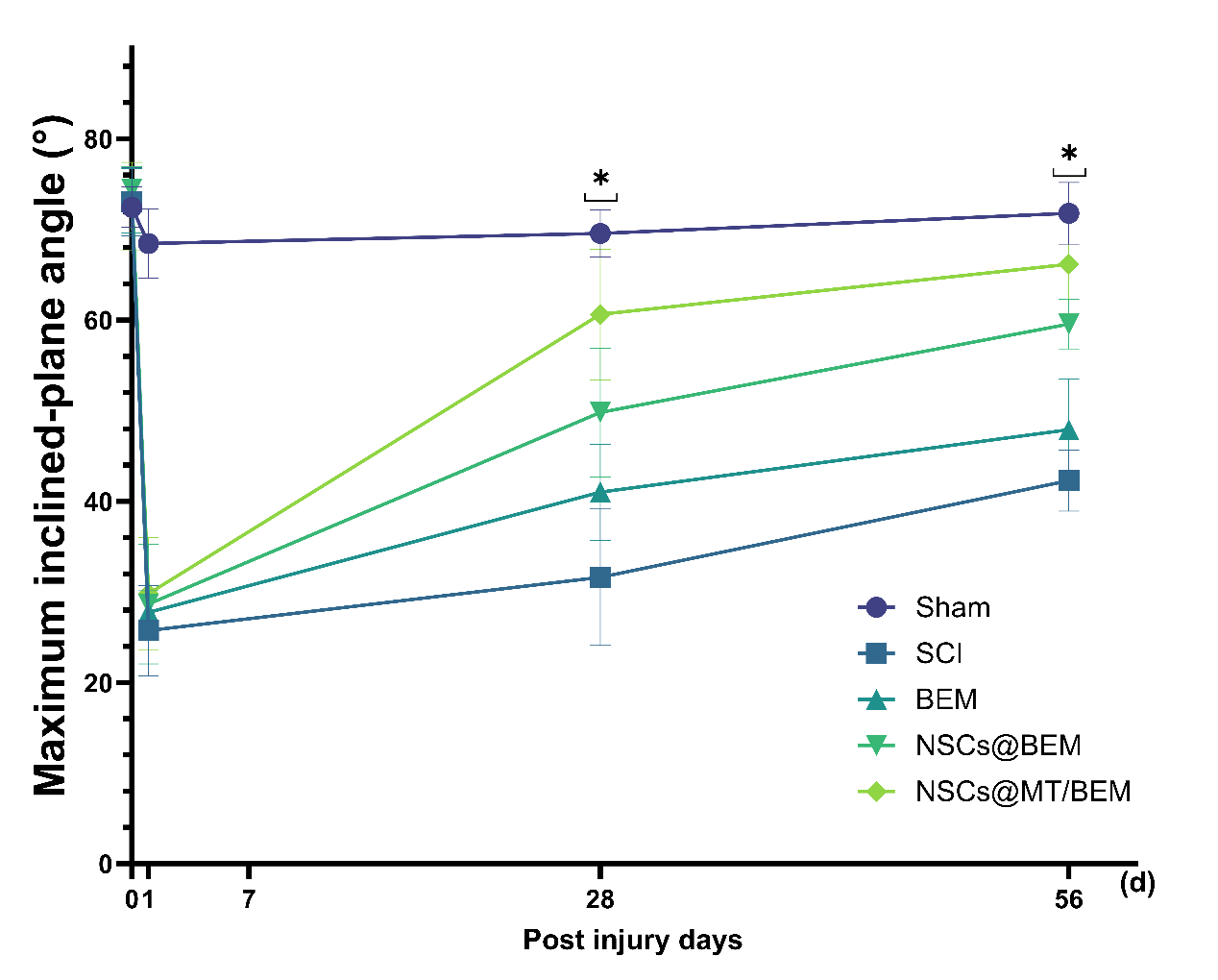


Figure S9. Maximum inclined-plane angle (°) at different time points post-injury. The graph shows the maximum inclined-plane angle achieved by each group at the indicated time points (Pre-injury, Post-injury days 1, 7, 28, and 56). Data are represented as mean ± SD (n = 10 per group). Statistical significance was determined using two-way repeated-measures ANOVA followed by Dunnett’s post-hoc test (vs SCI group). Asterisks indicate statistically significant differences: *p < 0.05.


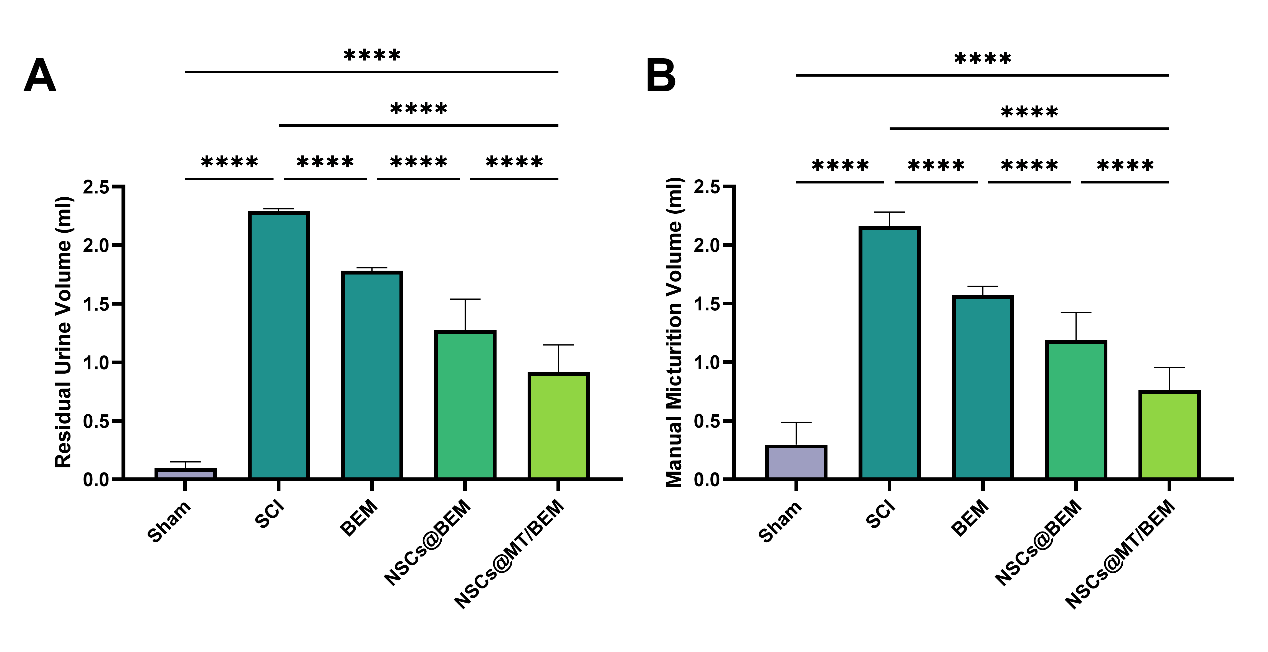


Figure S10. (A) Residual urine volume (PVR) measured 8 weeks post-injury showed a significant increase in residual urine volume in SCI rats. The NSCs@MT/BEM group exhibited the lowest residual urine volume compared to all treatment groups. (B) Manual micturition volume measured 8 weeks post-injury, with SCI rats demonstrating the highest manual micturition volume compared to the other groups. NSCs@MT/BEM treatment significantly improved bladder function in the rats. Data are presented as mean ± SD (n = 6). Statistical significance was determined by one-way ANOVA with Tukey's post-hoc test, with p < 0.0001 denoted by asterisks.


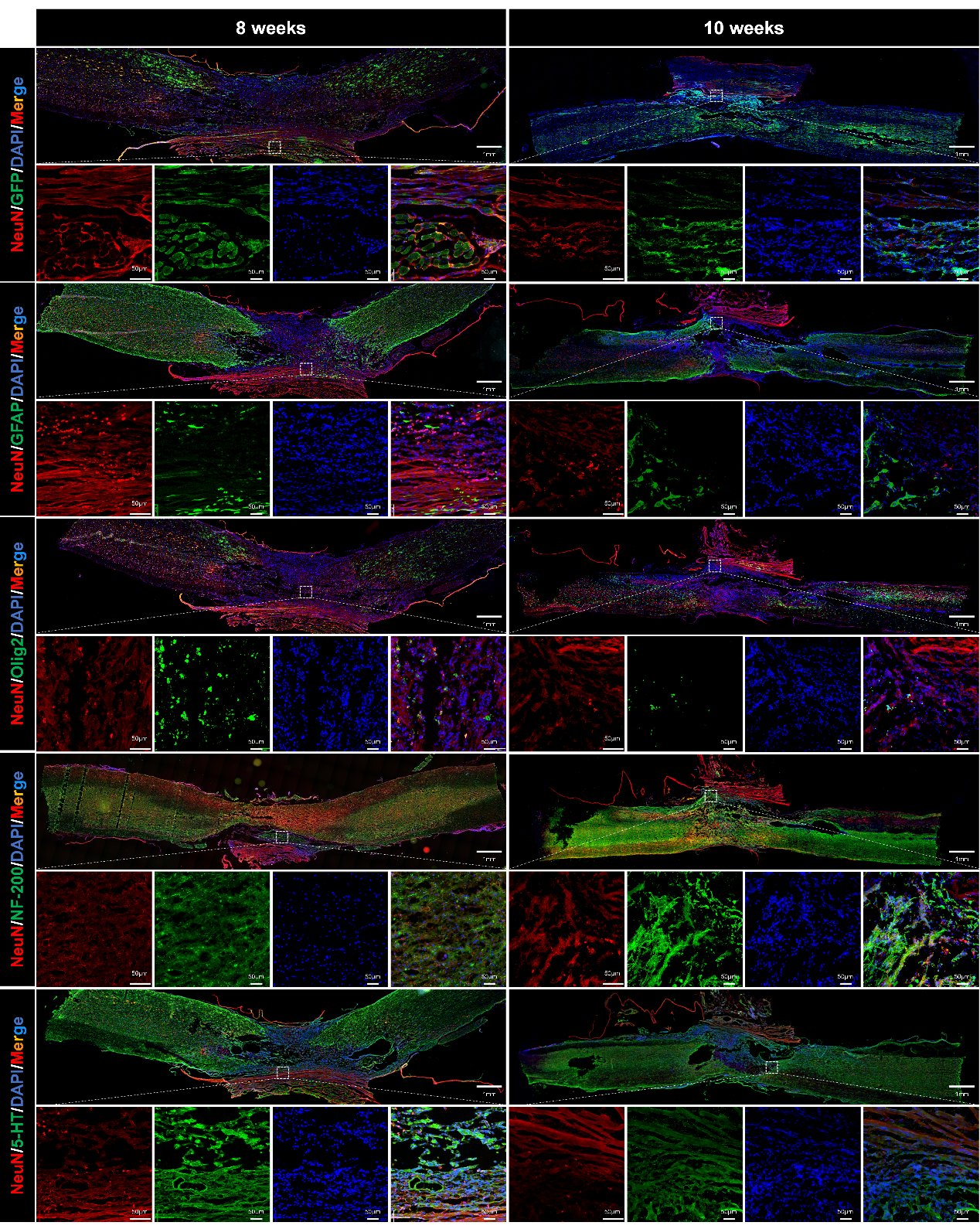
Figure S11. Representative comparative immunofluorescence images of the NSCs@MT/BEM group at 8 and 10 weeks after SCI.


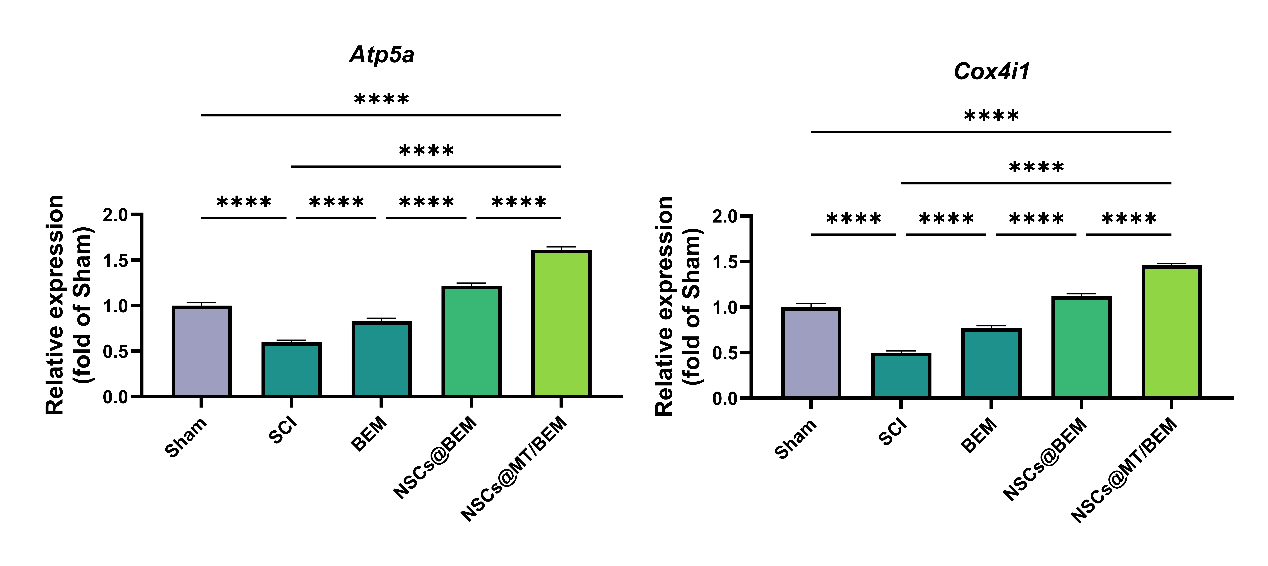


Figure S12. Relative mRNA expression of Cox4i1 and Atp5a1 in spinal cord tissue from the indicated treatment groups. *p < 0.05, **p < 0.01, ***p < 0.001, ****p < 0.0001.


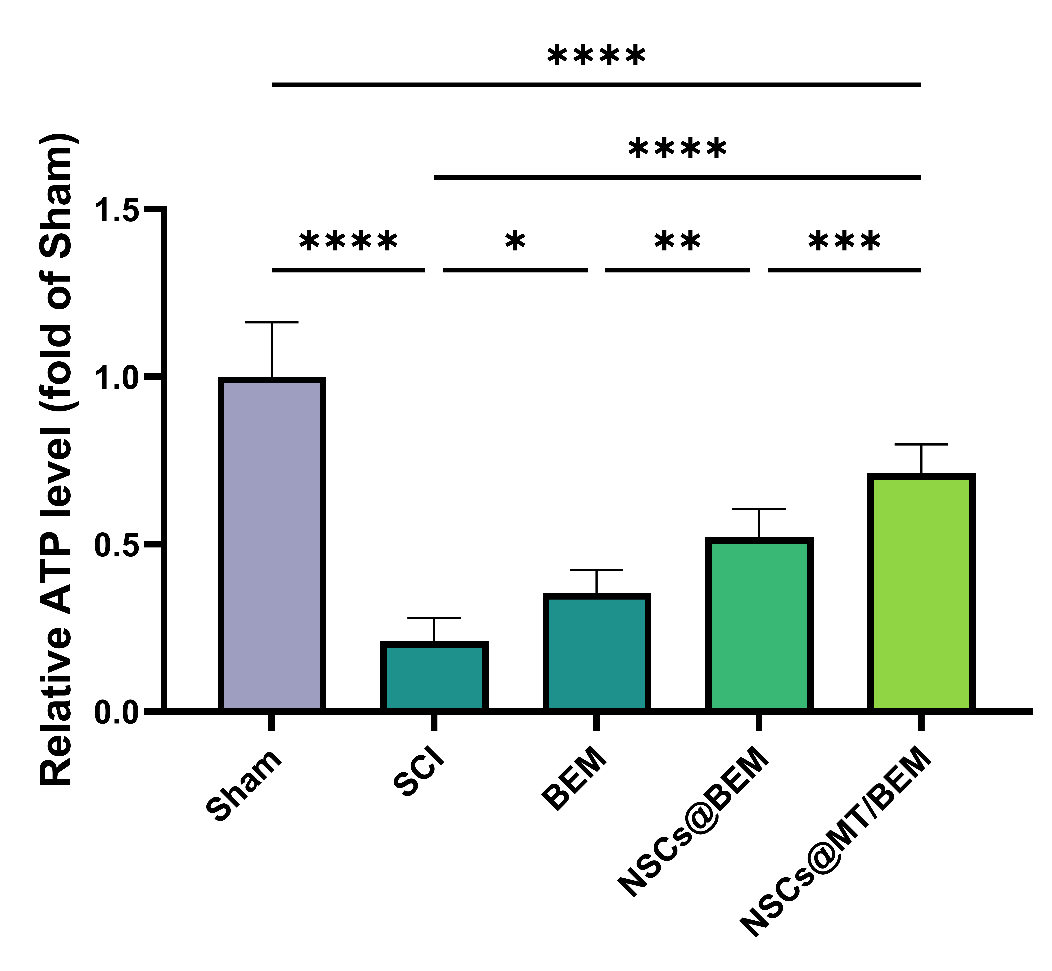
Figure S13. Spinal cord ATP levels at 4 weeks post-injury. ATP content in lesion-segment spinal cord tissue was quantified using a luciferase-based assay (Beyotime, S0026). Values were normalized to total protein and expressed as fold change versus Sham. Data are mean ± SD (n = 10/group). One-way ANOVA with Holm–Sidak’s multiple comparisons test; *p < 0.05, **p < 0.01, ***p < 0.001, ****p < 0.0001.

Table S1

|  | Forward primer | Reverse primer |
| --- | --- | --- |
| GFAP | TGGATCTGGAGAGGAAGGTTGAGTC | AGTTGGCGGCGATAGTCATTAGC |
| Olig2 | TCATCTTCCTCCAGCACCTCCTC | TCACCAGTCTCTTCATCTCCTCCAG |
| nestin | AGAAGAGGAGGACCAGAGGATTGTG | GCTCACTGCCATCTGCTCATTCC |
| β-tubulinⅢ | CGTCCACCTTCATCGGCAACAG | CTCCTCGTCGTCATCTTCATACATCTC |
| GAPDH | GTCCATGCCATCACTGCCACTC | CGCCTGCTTCACCACCTTCTTG |
| Prkaa1 | CTCGCCCAATTATGCTGCAC | TGGCACGTGGTCATCATCAA |
| Ppargc1a | CATGCAAACCACACCCACAG | CTGAGCAGGGACGTCTTTGT |
| Tfam | AATGTGGGGCGTGCTAAGAA | ACAGATAAGGCTGACAGGCG |
| Nrf1 | AGAGACAGCAGACACGGTTG | GCTGCGCCAAACACCTTAAA |
| Cox4i1 | GAGTGGAAGACAGTGGTGGG | GGATGGGGCCATACACGTAG |
| Atp5a1 | TGCCATTGATGGGAAGGGTC | TGGTTCCCGCACAGAGATTC |
| Mt-Nd1 | CCTAACATGACCACCCACCT | CCTTGAGTGCTTGTGGTGGA |
| Mt-Co1 | TGCAGGAGTAGGAGATAGGCA | GGTGATTGGTGTTGAGGTTG |
| Cpt1b | AGCCCCATCATGGTGAACAG | CCAGTTTGCGGCGATACATG |
| Pdk4 | TTGACATCCTCCCTGAACGC | GCTTTCTGGTCTTCTGGGCT |
| Ucp2 | GCAGTTCTACACCAAGGGCT | TGCTCTGGTATCTCCGACCA |
